# Supplementary material for: Molecular Structure of Salicylic Acid and Its Hydrates: A Rotational Spectroscopy Study
Source: Int J Mol Sci. 2024 Apr 6;25(7):4074. doi: 10.3390/ijms25074074 (PMC11012204; doi:10.3390/ijms25074074)
Supplement: Supplementary file 1 [file ijms-25-04074-s001.zip › ijms-2929731-supplementary.pdf]

Molecular Structure of Salicylic Acid and its Hydrates: A Rotational Spectroscopy Study

Supporting Information

Contents:

Determination of the θ angle

Figure S1. Rotational spectra of salicylic acid and its water complexes.

Figure S2. Predicted stable conformers of salicylic acid monomer.

Figure S3. Predicted stable conformers of the monohydrated complex of salicylic acid.

Figure S4. Predicted stable conformers of the dihydrated complex of salicylic acid.

Figure S5. Predicted stable conformers of the trihydrated complex of salicylic acid.

Figure S6. Predicted stable conformers of the tetrahydrated complex of salicylic acid.

Figure S7. Results of Quantum Theory of "atoms in molecules" (QTAIM) and non-covalent interaction (NCI) analyses for the observed conformer I of salicylic acid.

Figure S8. Results of Quantum Theory of "atoms in molecules" (QTAIM) and non-covalent interaction (NCI) analyses for the observed conformer I-w-a of the monohydrated complex of salicylic acid.

Figure S9. Results of Quantum Theory of "atoms in molecules" (QTAIM) and non-covalent interaction (NCI) analyses for the observed conformer I-w_2-a of the dihydrated cluster of salicylic acid.

Figure S10. Results of Quantum Theory of "atoms in molecules" (QTAIM) and non-covalent interaction (NCI) analyses for the observed conformer I-w_3-a of the trihydrated complex of salicylic acid.

Figure S11. Results of Quantum Theory of "atoms in molecules" (QTAIM) and non-covalent interaction (NCI) analyses for the observed conformer I-w_4-a of the tetrahydrated cluster of salicylic acid.

Figure S12. Possible path for inversion for the conformer I-w_3-a. It was calculated in two steps by scanning successively the flipping angles α and β. Through this path small potential energy barriers allow the interconversion between two equivalent forms. Calculations have been done at B3LYP-D3/6-311++G(d,p) level of theory.

Table S1. Rotational parameters predicted from B3LYP-D3/6-311++G(d,p) level of theory for the stable conformers of salicylic acid monomer.

Table S2. Rotational parameters predicted from B3LYP-D3/6-311++G(d,p) level of theory for the stable conformers of the monohydrated complex of salicylic acid.

Table S3. Rotational parameters predicted from B3LYP-D3/6-311++G(d,p) level of theory for the stable conformers of the dihydrated complex of salicylic acid.

Table S4. Rotational parameters predicted from B3LYP-D3/6-311++G(d,p) level of theory for the stable conformers of the trihydrated complex of salicylic acid.

Table S5. Rotational parameters predicted from B3LYP-D3/6-311++G(d,p) level of theory for the stable conformers of the tetrahydrated complex of salicylic acid.

Table S6. Rotational parameters experimentally determined for the for all the observed ^{13}C and D isotopologues of conformer I of salicylic acid.

Table S7. Rotational parameters experimentally determined for all the observed D isotopologues of conformer I-w-a of salicylic acid – water cluster.

Table S8. r_s, r_m, r_a and r_e geometrical parameters for conformer I of salicylic acid.

Table S9. r_s, r_m and r_e coordinates determined for conformer I of salicylic acid.

Table S10. Donor-acceptor stabilizing energies predicted from NBO calculations at B3LP-D3/6-311++G(d,p) level of theory for all the observed I, I-w-a, I-w_2-a, I-w_3-a and I-w_4-a species of the salicylic acid and its hydrated complexes.

Table S11. Bond orders predicted from NBO calculations at B3LP-D3/6-311++G(d,p) level of theory for all the observed I, I-w-a, I-w_2-a, I-w_3-a and I-w_4-a species of the salicylic acid and its hydrated complexes.

Table S12. r_s, r_0 and r_e geometrical parameters determined for conformer I-w-a of the monohydrated complex of salicylic acid.

Table S13. r_s, r_0 and r_e coordinates determined for conformer I-w-a of the monohydrated complex of salicylic acid.

Table S14. r_e geometrical parameters predicted for conformer I-w_2-a of the dihydrated complex of salicylic acid.

Table S15. r_e geometrical parameters predicted for conformer I-w_3-a of the trihydrated complex of salicylic acid.

Table S16. r_e geometrical parameters predicted for conformer I-w_4-a of the tetrahydrated complex of salicylic acid.

Table S17. Transition frequencies of conformer I of salicylic acid monomer.

Table S18. Transition frequencies of all the observed ^{13}C isotopologues of conformer I of salicylic acid monomer.

Table S19. Transition frequencies of all the observed D isotopologues of conformer I of salicylic acid monomer.

Table S20. Transition frequencies of conformer I-w-a of salicylic acid – water complex.

Table S21. Transition frequencies of all the observed D isotopologues of conformer I-w-a of salicylic acid – water complex.

Table S22. Transition frequencies of conformer I-w_2-a of salicylic acid – $water_2$ complex.

Table S23. Transition frequencies of conformer I-w_3-a of salicylic acid – $water_2$ complex.

Table S24. Transition frequencies of conformer I-w_4-a of salicylic acid – $water_2$ complex.

Determination of the θ angle

For monohydrated complexes with a planar or nearly planar structure, in which the *ab* inertial plane roughly overlaps with the same inertial plane of the monomer form, Ouyang and Howard [31] developed a method in order to determine the angle θ between the *a* principal axis of the monomer form and the intermolecular axis of the cluster using the next expression:

$$sin^2\theta = \frac{I_A(\mu R_{CM}^2 + I_a + I_b - I_A) - I_a(\mu R_{CM}^2 + I_b)}{\mu R_{CM}^2(I_b - I_a)} \quad (1)$$

where I_A, I_B and I_C are the principal moments of inertia of the monohydrated complex while I_a, I_b and I_c are those of the monomer form, μ is the reduced mass considering a pseudo-diatomic model and R_{CM} is the distance between the centers of mass of the monomer and the water molecule in the complex. R_{CM} could be taken from the geometrical predictions or calculated by the equation:

$$R_{CM}^2 = \frac{(I_C - I_c)}{\mu} \quad (2)$$

Figure S1. CP-FTMW, 2-8 GHz, rotational spectrum of SA and its water complexes (upper figure). At the bottom, a zoom of the spectrum form 6-8 GHz is shown, where the intensities of the most intense transitions of the SA-I and SA-w (SA-I-w-a) species can be compared. The a) excerpt shows the $4_{0,4} \leftarrow 3_{1,3}$ transition for the SA-I ^{13}C isotopologues. (See Figure 3 for the atom labelling).

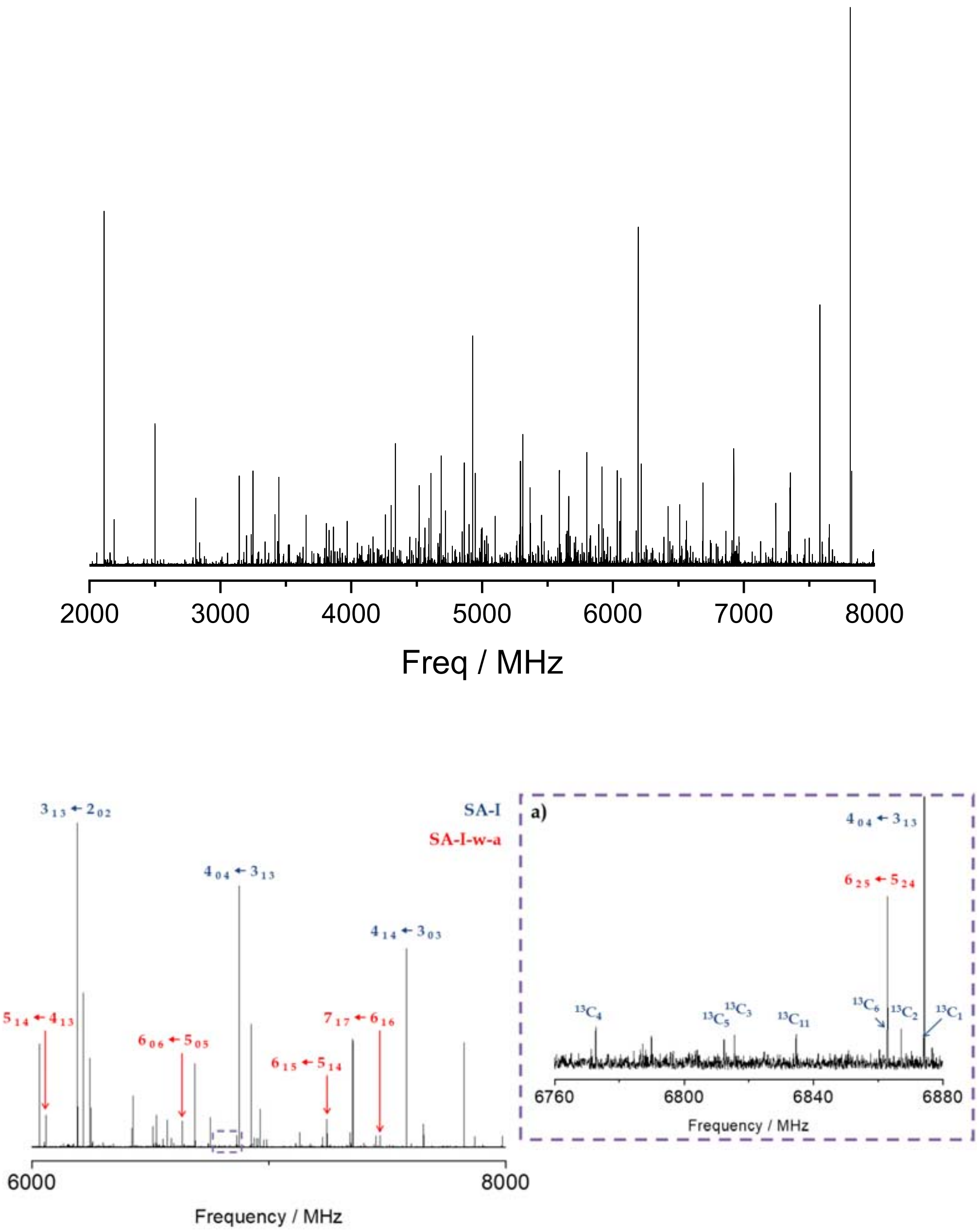

Figure S2. Stable conformers predicted for salicylic acid monomer. Their corresponding parameters are given in Table S1. Relative energies to conformer I are predicted at B3LYP-D3-6-311++G(d,p) level.

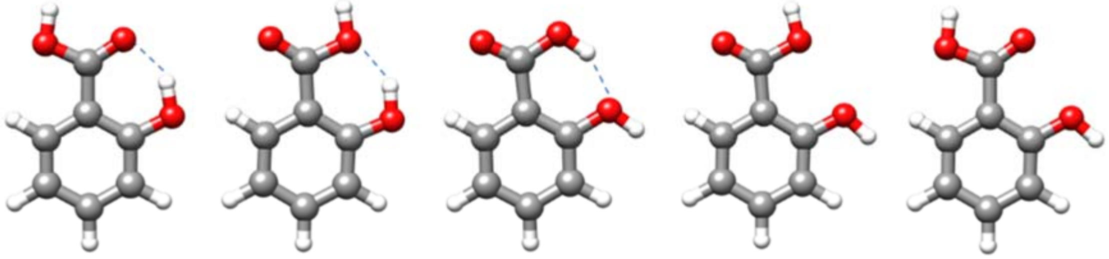

I
0.0 cm^{-1}

II
1144.6 cm^{-1}

III
3365.1 cm^{-1}

IV
3786.0 cm^{-1}

V
3913.2 cm^{-1}

Figure S3. Stable conformers predicted for the monohydrated complex of salicylic acid. Their corresponding parameters are given in Table S2. Relative energies to conformer I-w-a are predicted at B3LYP-D3-6-311++G(d,p) level.

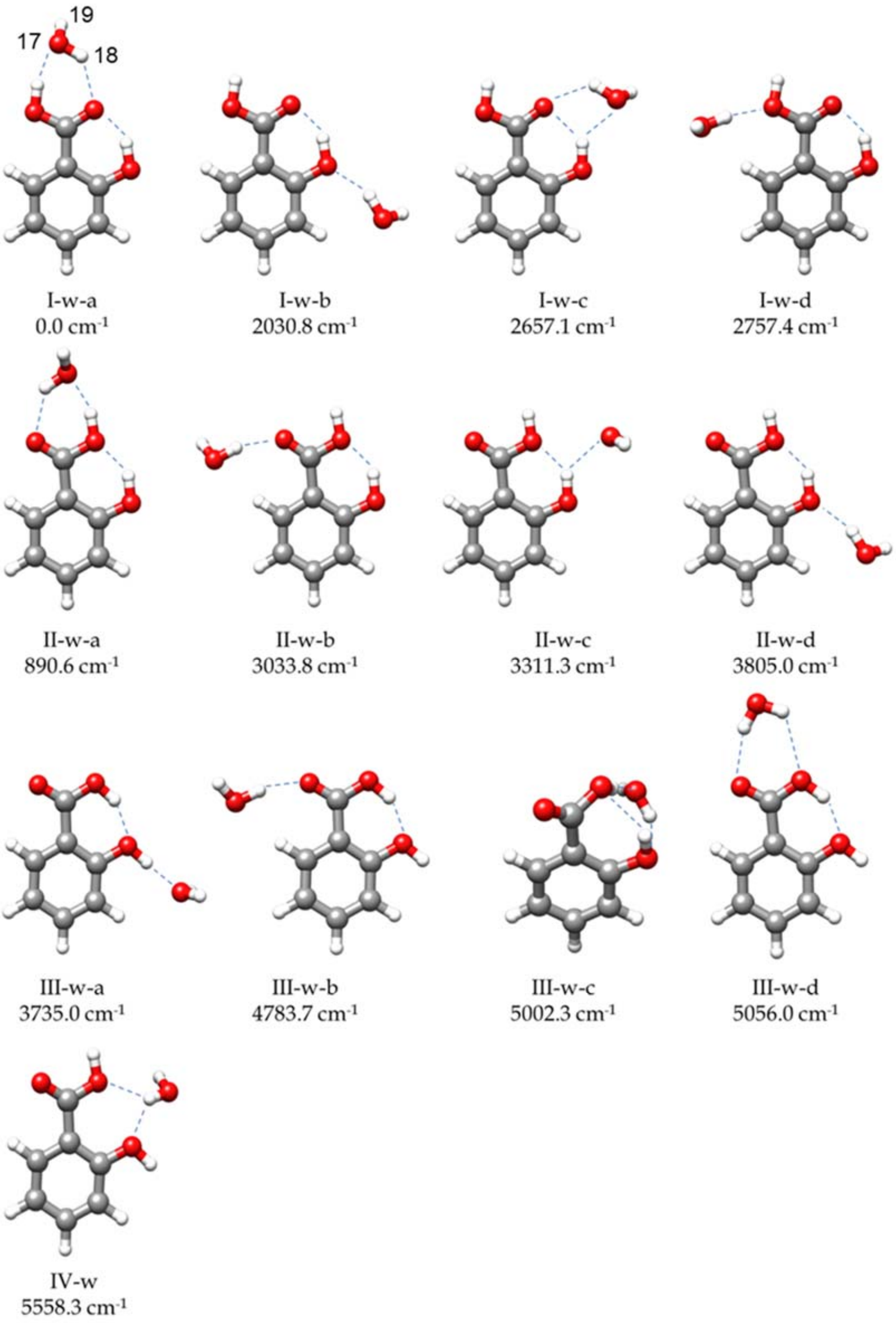

Figure S4. Stable conformers predicted for the dihydrated complex of salicylic acid. The parameters for the first-row conformers are given in Table S3. Relative energies to conformer I-w2-a are predicted at B3LYP-D3-6-311++G(d,p) level.

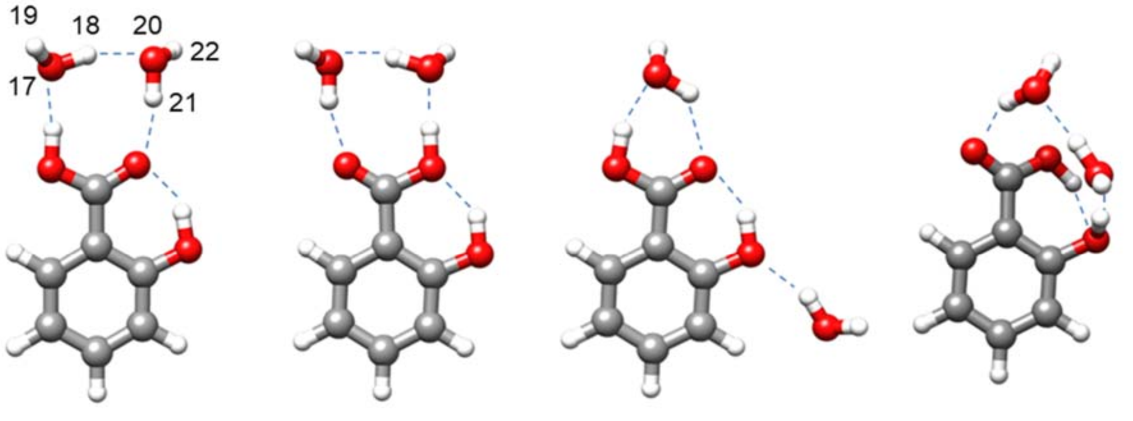

I-w$_2$-a
0.0 cm^{-1}

II-w$_2$-a
693.9 cm^{-1}

I-w$_2$-ab
2401.6 cm^{-1}

III-w$_2$-a
3575.7 cm^{-1}

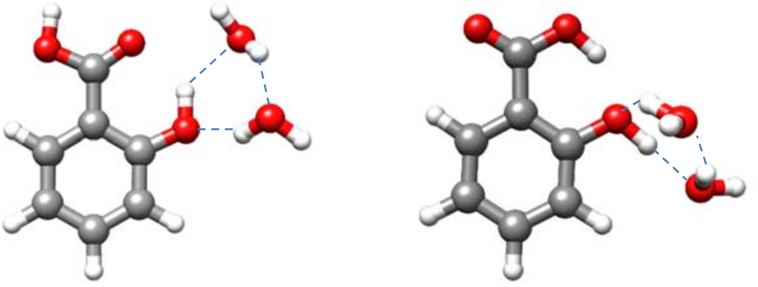

I-w$_2$-ph
3291 cm^{-1}

III-w$_2$-ph
4543 cm^{-1}

Figure S5. Stable conformers predicted for the trihydrated complex of salicylic acid. The parameters are given in Table S4. Relative energies to conformer I-w3-a are predicted at B3LYP-D3/6-311++G(d,p) level.

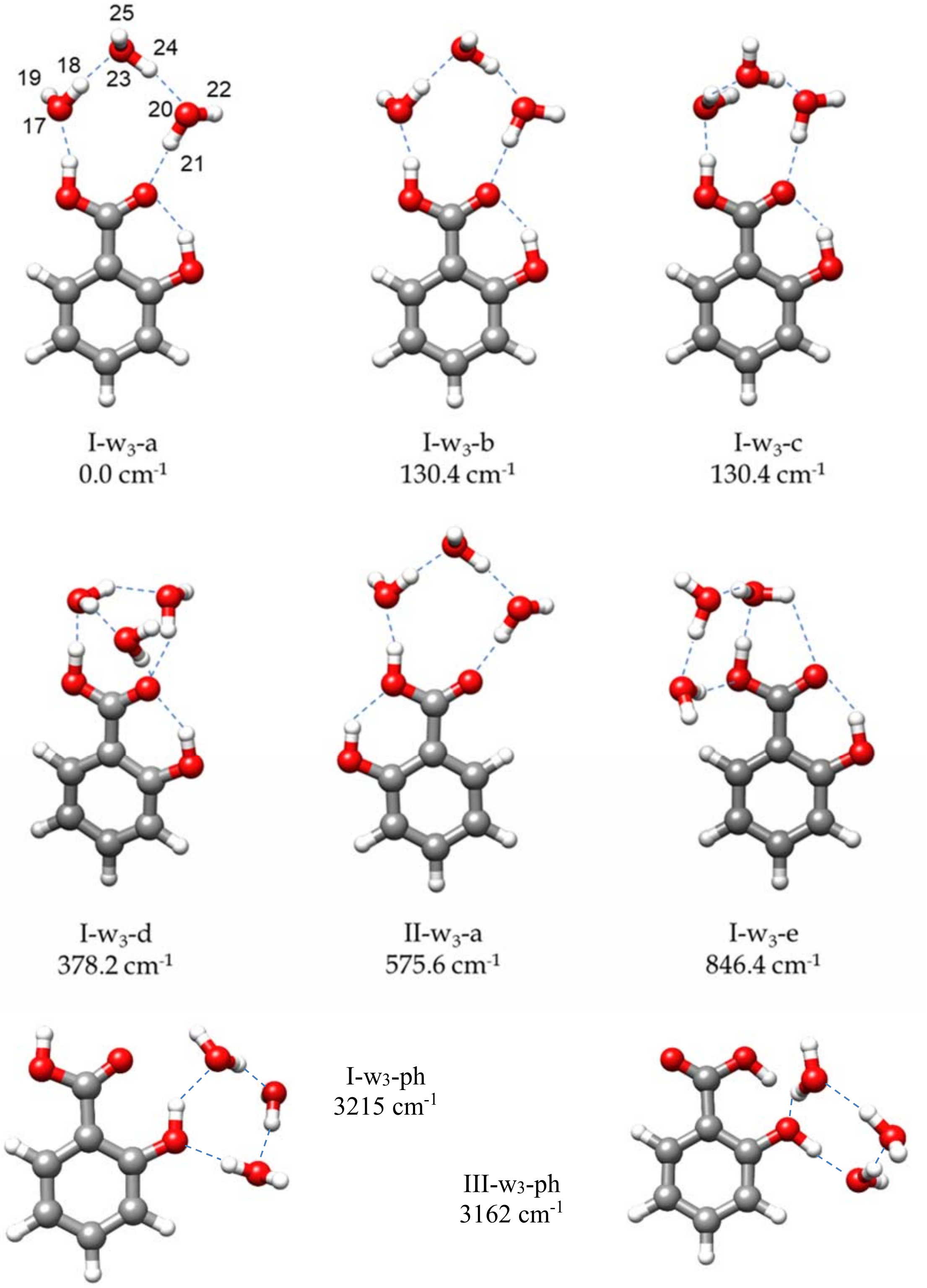

Figure S6. Stable conformers predicted for the tetrahydrated complex of salicylic acid. Their corresponding parameters are given in Table S 5. Relative energies to conformer I-w4-a are predicted at B3LYP-D3/6-311++G(d,p) level.

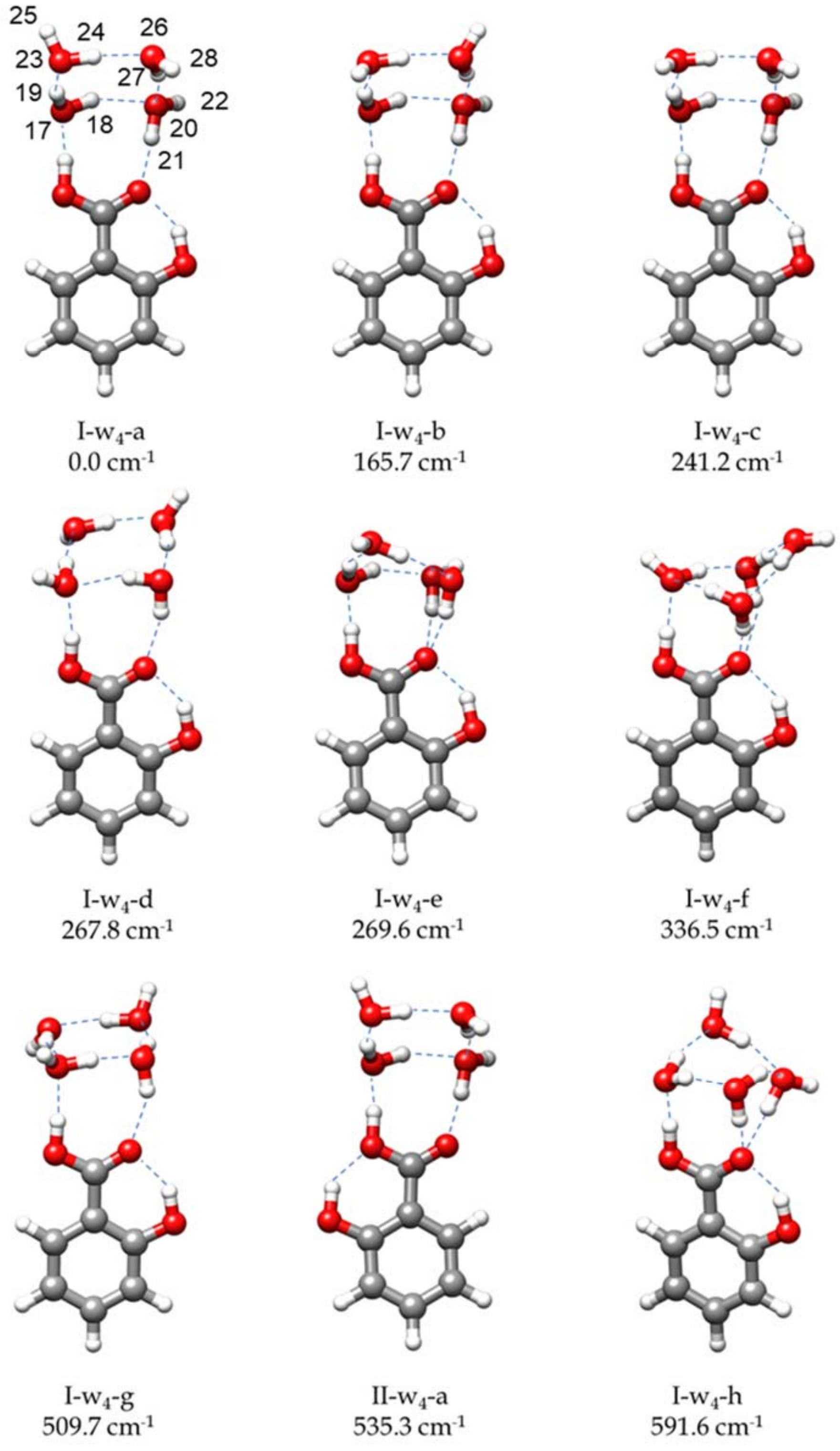

Figure S7. a) Bond Critical Points (BCPs) and Ring Critical Points (RCPs) in yellow, and the Bond Paths (BPs) in orange calculated at B3LYP-D3/6-311++G(d,p) level for the conformer I of salicylic acid from the Quantum Theory of "atoms in molecules" (QTAIM) analyses. b) Results of Non-Covalent Interaction (NCI) analysis. Each point in the scatter graph corresponds to a grid point in 3D space and represents the reduced density gradient (RDG) vs. sign[$\lambda_2(r)$]$\rho(r)$. The blue-green spikes in the left (negative side) corresponds to attractive interactions, while those orange-red spikes in the right (positive side) corresponds to negative interactions. The points corresponding to low RDG values represent weak interactions. Those with RDG<0.5 a.u. are represented by the isosurface.

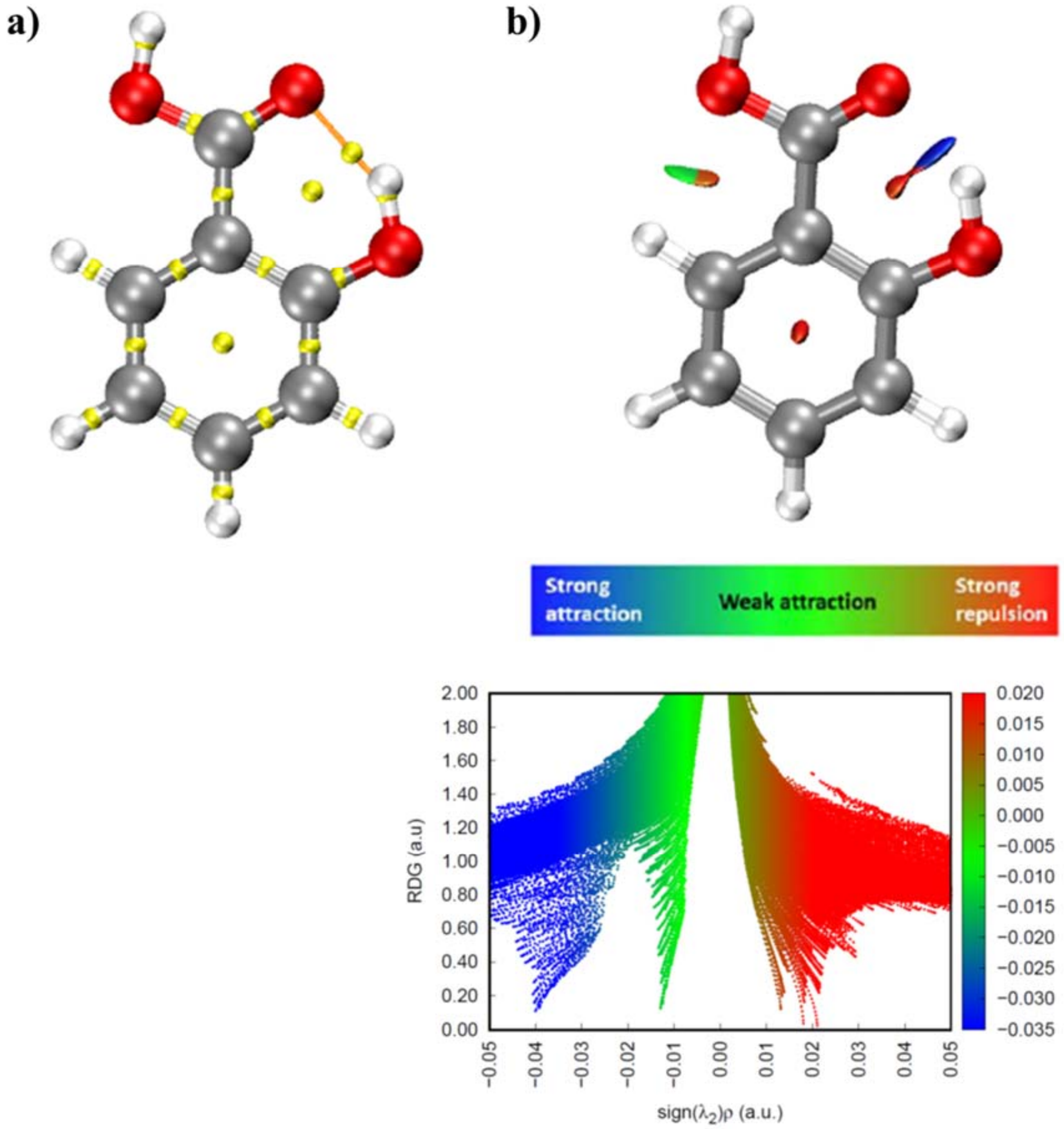

Figure S8. a) Bond Critical Points (BCPs) and Ring Critical Points (RCPs) in yellow, and the Bond Paths (BPs) in orange calculated at B3LYP-D3/6-311++G(d,p) level for the conformer I-w-a of salicylic acid – water complex from the Quantum Theory of "atoms in molecules" (QTAIM) analyses. b) Results of Non-Covalent Interaction (NCI) analysis. Each point in the scatter graph corresponds to a grid point in 3D space and represents the reduced density gradient (RDG) vs. sign[$\lambda_2(r)$]$\rho(r)$. The blue-green spikes in the left (negative side) corresponds to attractive interactions, while those orange-red spikes in the right (positive side) corresponds to negative interactions. The points corresponding to low RDG values represent weak interactions. Those with RDG<0.5 a.u. are represented by the isosurface.

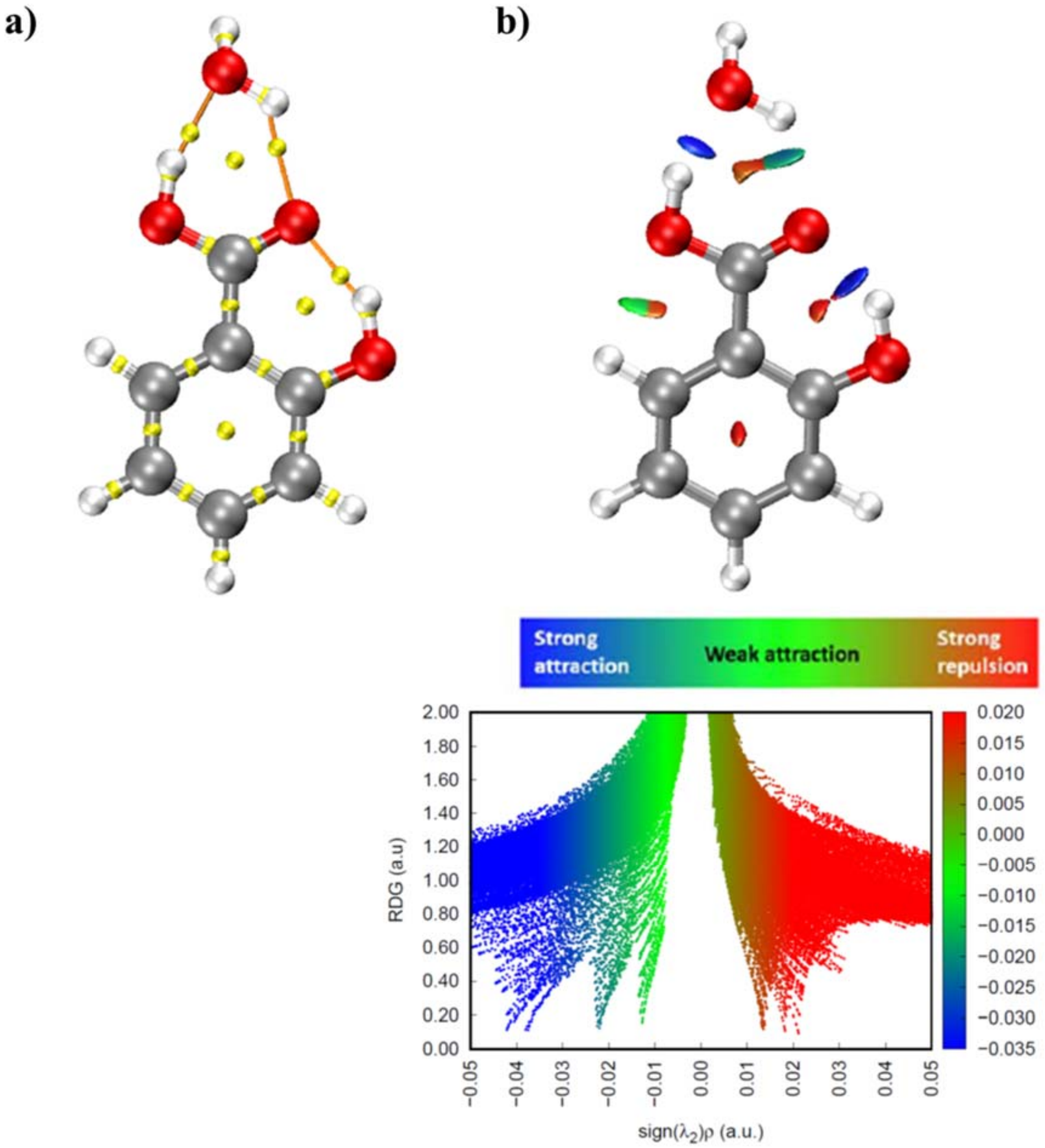

Figure S9. a) Bond Critical Points (BCPs) and Ring Critical Points (RCPs) in yellow, and the Bond Paths (BPs) in orange calculated at B3LYP-D3/6-311++G(d,p) level for the conformer I-w$_2$-a of salicylic acid – water$_2$ complex from the Quantum Theory of "atoms in molecules" (QTAIM) analyses. b) Results of Non-Covalent Interaction (NCI) analysis. Each point in the scatter graph corresponds to a grid point in 3D space and represents the reduced density gradient (RDG) vs. sign[$\lambda_2(r)$]$\rho(r)$. The blue-green spikes in the left (negative side) corresponds to attractive interactions, while those orange-red spikes in the right (positive side) corresponds to negative interactions. The points corresponding to low RDG values represent weak interactions. Those with RDG<0.5 a.u. are represented by the isosurface.

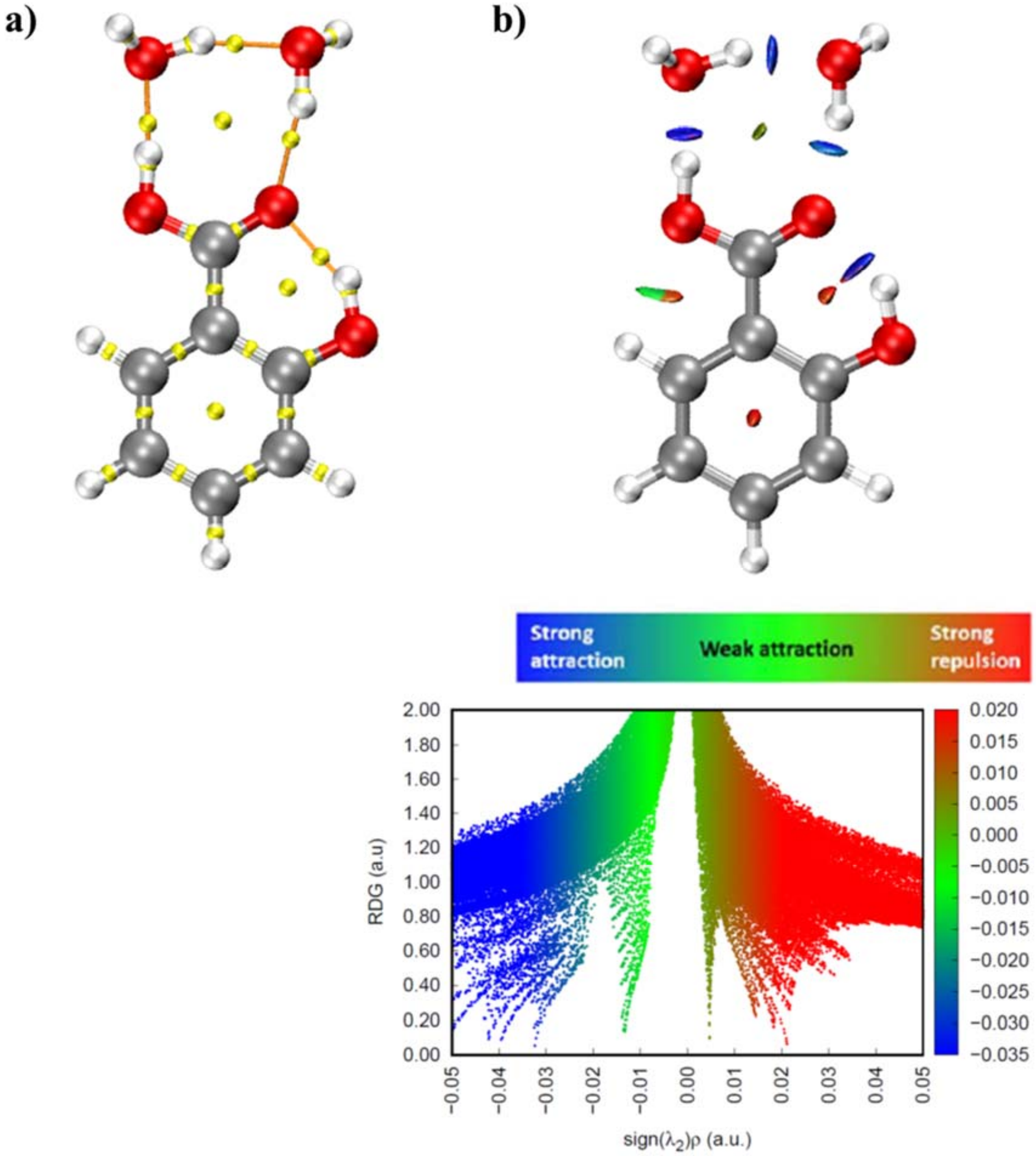

Figure S10. a) Bond Critical Points (BCPs) and Ring Critical Points (RCPs) in yellow, and the Bond Paths (BPs) in orange calculated at B3LYP-D3/6-311++G(d,p) level for the conformer I-w$_3$-a of salicylic acid – water$_3$ complex from the Quantum Theory of "atoms in molecules" (QTAIM) analyses. b) Results of Non-Covalent Interaction (NCI) analysis. Each point in the scatter graph corresponds to a grid point in 3D space and represents the reduced density gradient (RDG) vs. sign[$\lambda_2(r)$]$\rho(r)$. The blue-green spikes in the left (negative side) corresponds to attractive interactions, while those orange-red spikes in the right (positive side) corresponds to negative interactions. The points corresponding to low RDG values represent weak interactions. Those with RDG<0.5 a.u. are represented by the isosurface.

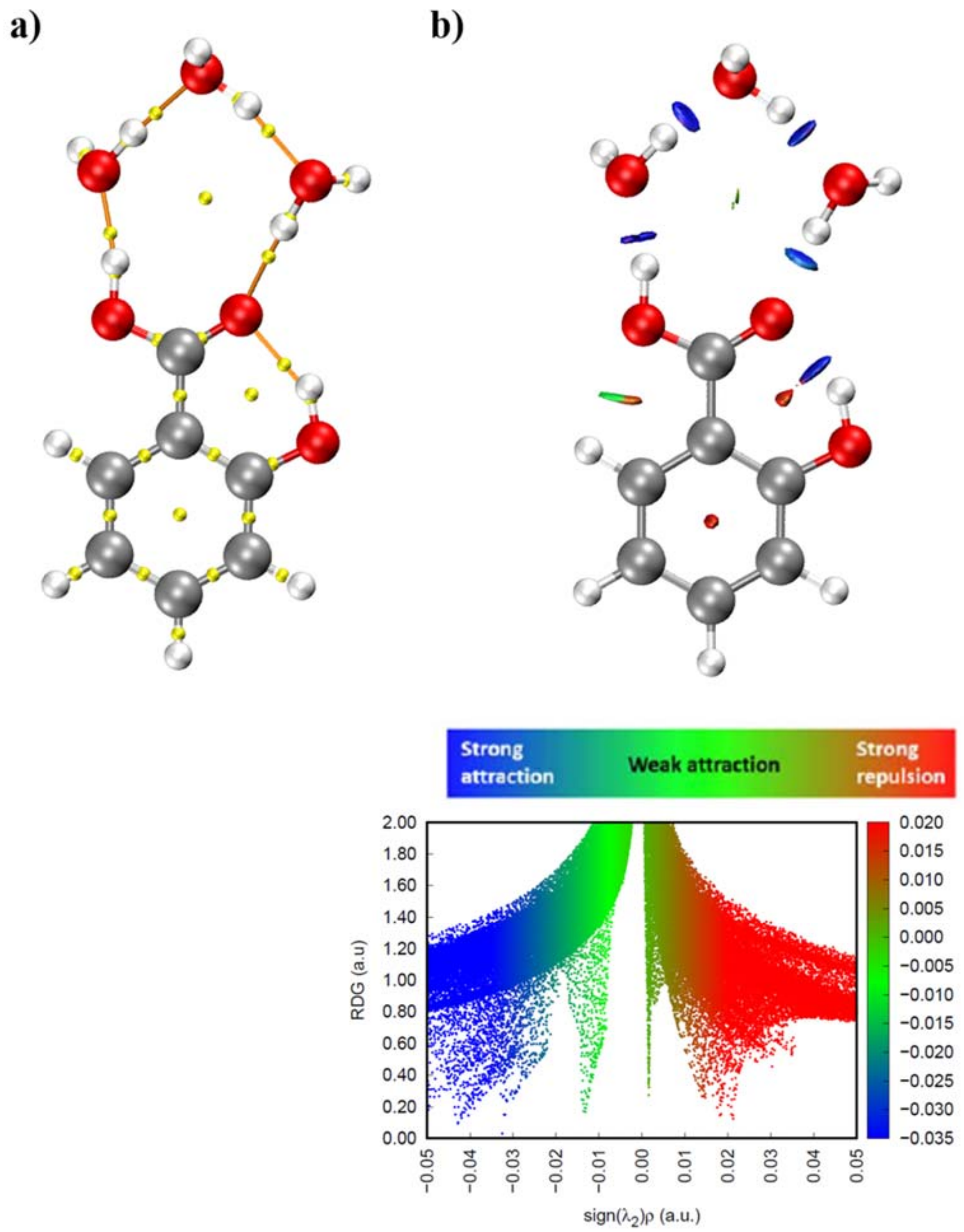

Figure S11. a) Bond Critical Points (BCPs) and Ring Critical Points (RCPs) in yellow, and the Bond Paths (BPs) in orange calculated at B3LYP-D3/6-311++G(d,p) level for the conformer I-w_4-a of salicylic acid – $water_4$ complex from the Quantum Theory of "atoms in molecules" (QTAIM) analyses. b) Results of Non-Covalent Interaction (NCI) analysis. Each point in the scatter graph corresponds to a grid point in 3D space and represents the reduced density gradient (RDG) vs. sign[$\lambda_2(r)$]$\rho(r)$. The blue-green spikes in the left (negative side) corresponds to attractive interactions, while those orange-red spikes in the right (positive side) corresponds to negative interactions. The points corresponding to low RDG values represent weak interactions. Those with RDG<0.5 a.u. are represented by the isosurface. The hydrogen bond between the O-H···Ow_1 presents a sign[$\lambda_2(r)$]$\rho(r)$ value close to -0.007 a.u., associated to a strong interaction.

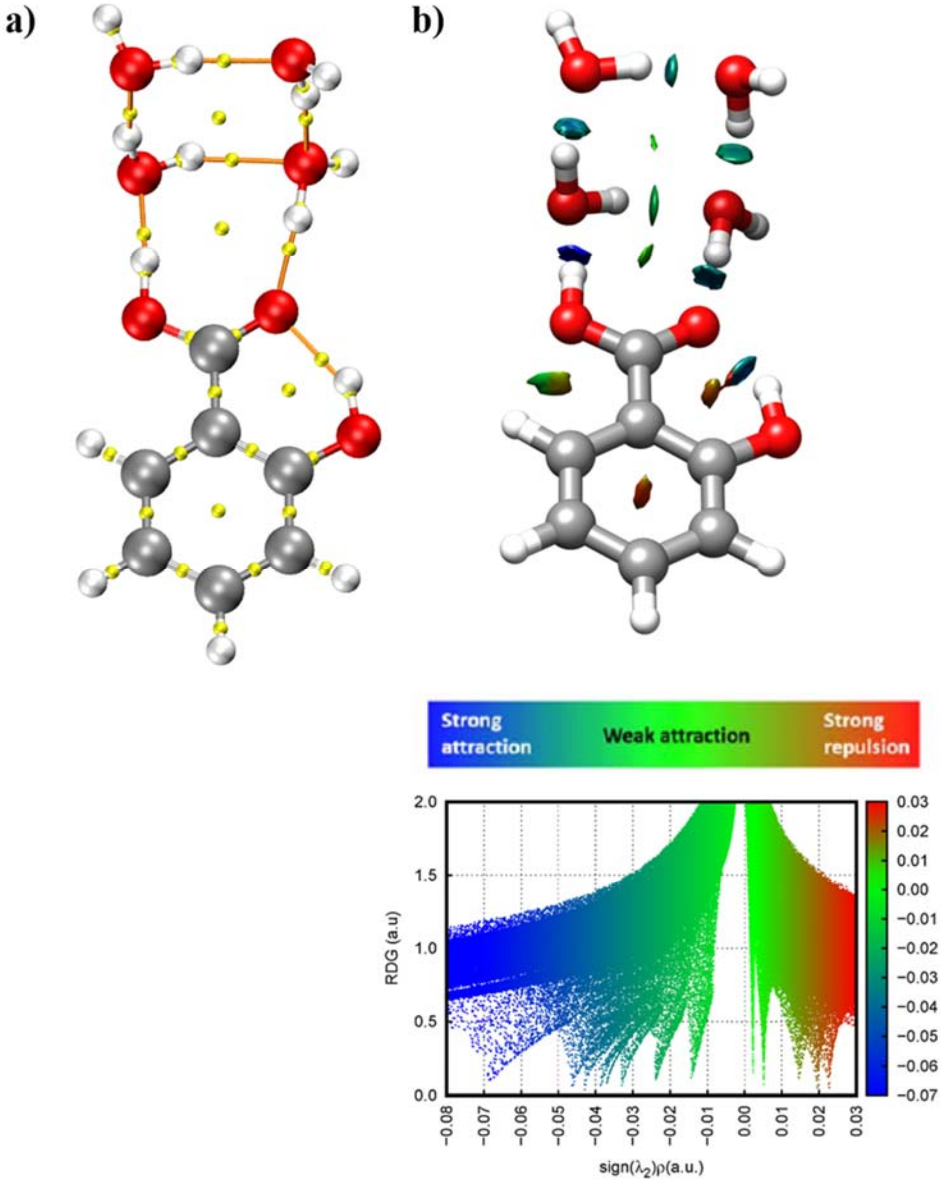

Figure S12. Possible path for inversion for the conformer I-w3-a. It was calculated in two steps by scanning successively the flipping angles α and β. Through this path small potential energy barriers allow the interconversion between two equivalent forms. Calculations have been done at B3LYP-D3/6-311++G(d,p) level of theory.

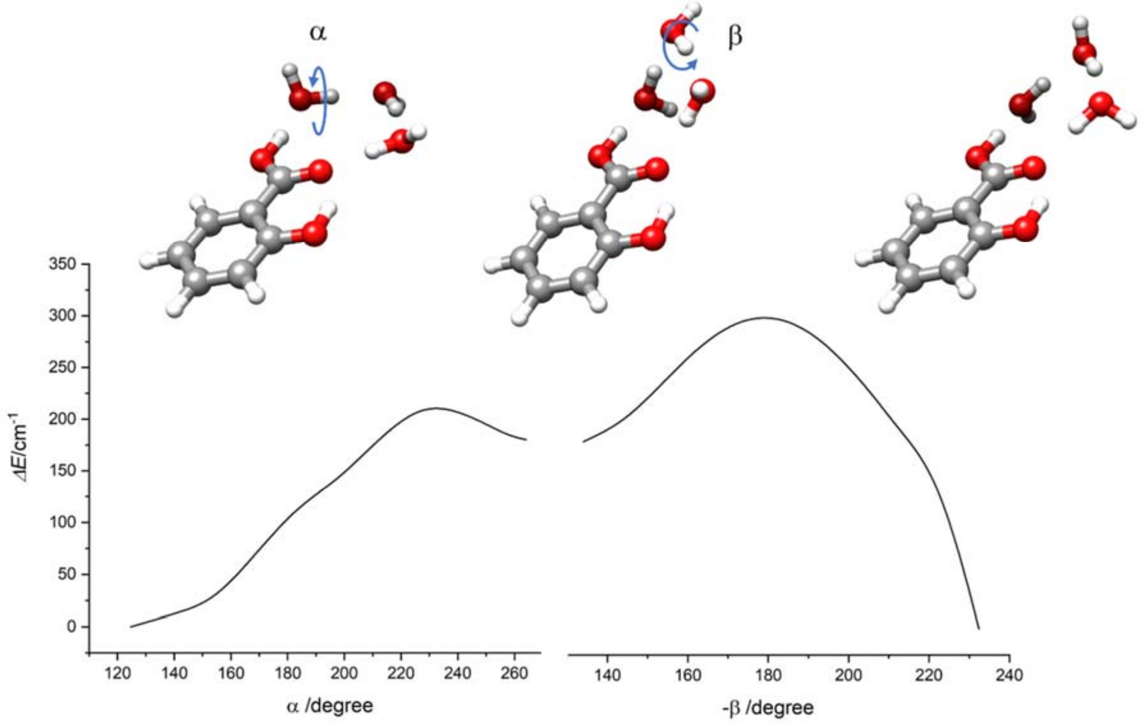

Table S1. Rotational parameters predicted from B3LYP-D3/6-311++G(d,p) level of theory for the most stable species of salicylic acid monomer.

**Parameter** [a]	**I**	**II**	**III**	**IV**	**V**
A/MHz	2336.06	2329.26	2322.93	2318.42	2295.69
B/MHz	1221.82	1211.10	1208.39	1205.87	1210.29
C/MHz	802.23	796.80	795.29	793.85	792.49
P_{cc}/uÅ^2	0.00	0.00	0.16	0.23	0.00
μ_a/D	-0.4	-0.1	5.7	2.8	2.4
μ_b/D	2.2	-0.7	3.7	2.2	-1.9
μ_c/D	0.0	0.0	0.3	0.1	0.0
ΔE_{DFT}/cm^{-1}	0.0 [b]	1144.6	3365.1	3786.0	3913.2

[a] A, B and C are the rotational constants. P_{cc} is the planar moment of inertia, derived from $P_{cc}=(I_a+I_b-I_c)/2$. μ_a, μ_b and μ_c are the components of the electric dipole moment. ΔE is the energy relative to predicted global minimum conformer.

[b] Absolute energy is -496.2168409 E_h.

Table S2. Rotational parameters predicted at B3LYP-D3/6-311++G(d,p) level of theory for the stable conformers of the monohydrated complex of the salicylic acid.

Parameter[a]	I-w-a	II-w-a	I-w-b	I-w-c	I-w-d
A/MHz	2313.15	2311.93	1399.25	1581.85	1335.12
B/MHz	648.76	647.51	821.10	805.29	870.84
C/MHz	507.09	506.29	517.80	534.31	537.91
P_{cc}/uÅ^2	0.42	0.44	0.33	0.60	9.67
μ_a/D	-0.6	-0.7	-2.5	0.4	0.3
μ_b/D	1.0	-0.6	0.5	2.0	0.6
μ_c/D	-1.2	1.2	0.9	1.3	1.1
ΔE_{DFT}/cm^{-1}	0.0 [b]	890.6	2030.8	2657.1	2757.4
	**II-w-b**	**II-w-c**	**III-w-a**	**II-w-d**	**III-w-b**
A/MHz	1355.92	1379.21	1223.98	1527.73	1348.17
B/MHz	868.96	816.33	842.59	790.54	878.31
C/MHz	529.99	513.19	516.33	522.23	532.79
P_{cc}/uÅ^2	0.37	0.37	16.95	1.18	0.85
μ_a/D	-0.6	0.4	9.4	1.7	6.2
μ_b/D	1.8	-1.3	1.7	-2.8	-0.5
μ_c/D	1.0	-1.1	0.2	0.0	1.0
ΔE_{DFT}/cm^{-1}	3033.8	3311.3	3735.0	3805.0	4783.7
	**III-w-c**	**III-w-d**	**IV-w**		
A/MHz	1361.27	2276.47	1425.13		
B/MHz	968.06	605.99	836.89		
C/MHz	688.29	478.71	549.88		
P_{cc}/uÅ^2	79.53	0.13	19.71		
μ_a/D	3.3	9.3	2.3		
μ_b/D	-4.0	3.6	-1.6		
μ_c/D	2.3	0.2	0.8		
ΔE_{DFT}/cm^{-1}	5002.3	5056.0	5558.3		

[a] See footnote of Table S1.

[b] Absolute energy is -572.6949094 E_h.

Table S3. Rotational parameters predicted at B3LYP-D3/6-311++G(d,p) level of theory for the stable conformers of the dihydrated complex of the salicylic acid.

Parameter[a]	I-2wa	II-2wa	I-2wab	III-2wa
A/MHz	1780.40	1759.99	1164.04	1385.97
B/MHz	453.67	450.32	526.32	613.62
C/MHz	362.04	359.17	362.96	504.06
P_{cc}/uÅ^2	0.96	1.17	0.99	92.81
μ_a/D	-0.5	0.8	-2.6	-1.4
μ_b/D	-0.9	-0.8	0.2	1.2
μ_c/D	0.0	0.0	2.2	-1.1
ΔE_{DFT}/cm^{-1}	0.0 [b]	693.9	2401.6	3571.7

[a] See footnote of Table S1.

[b] Absolute energy is -649.1747737 E_h.

Table S4. Rotational parameters predicted at B3LYP-D3/6-311++G(d,p) level of theory for the stable conformers of the trihydrated complex of the salicylic acid.

**Parameter**[a]	**I-w3-a**	**I-w3-b**	**I-w3-c**
A/MHz	1412.10	1436.05	1421.44
B/MHz	319.03	317.16	327.39
C/MHz	263.04	262.98	284.18
P_{cc}/uÅ^2	10.33	11.82	60.41
μ_a/D	0.6	-0.1	-1.5
μ_b/D	0.6	0.0	1.3
μ_c/D	0.6	0.3	-0.1
ΔE_{DFT}/cm^{-1}	0.0 [b]	130.4	130.4
	**I-w3-d**	**II-w3-a**	**I-w3-e**
A/MHz	1299.61	1352.11	1155.81
B/MHz	372.95	320.69	379.40
C/MHz	335.34	261.57	318.21
P_{cc}/uÅ^2	118.44	8.79	90.55
μ_a/D	1.1	0.9	-0.1
μ_b/D	-1.4	1.2	3.0
μ_c/D	1.2	0.8	1.0
ΔE_{DFT}/cm^{-1}	378.2	575.6	846.4

[a] See footnote of Table S1.

[b] Absolute energy is -725.6502119 E_h.

Table S5. Rotational parameters predicted at B3LYP-D3/6-311++G(d,p) level of theory for the stable conformers of the tetrahydrated complex of the salicylic acid.

**Parameter**[a]	**I-w$_4$-a**	**I-w$_4$-b**	**I-w$_4$-c**
A/MHz	1073.10	1066.29	1059.87
B/MHz	250.60	251.56	251.90
C/MHz	222.98	224.02	224.40
P_{cc}/uÅ^2	110.58	113.49	115.48
μ_a/D	-0.8	-0.7	0.8
μ_b/D	1.4	1.6	1.3
μ_c/D	0.2	0.4	1.6
ΔE_{DFT}/cm^{-1}	0.0 [b]	165.7	241.2
	**I-w$_4$-d**	**I-w$_4$-e**	**I-w$_4$-f**
A/MHz	1093.11	1028.22	1137.26
B/MHz	243.76	289.50	276.83
C/MHz	217.21	261.57	244.94
P_{cc}/uÅ^2	104.46	152.55	130.35
μ_a/D	0.6	-1.4	-1.3
μ_b/D	-3.1	0.1	-1.5
μ_c/D	0.5	0.5	0.1
ΔE_{DFT}/cm^{-1}	267.8	269.6	336.5
	**I-w$_4$-g**	**II-w$_4$-a**	**I-w$_4$-h**
A/MHz	1045.06	1062.27	1053.34
B/MHz	251.42	251.68	288.32
C/MHz	227.64	222.95	260.13
P_{cc}/uÅ^2	136.80	112.50	144.92
μ_a/D	0.4	1.1	-0.1
μ_b/D	-3.1	3.0	1.6
μ_c/D	0.3	-0.2	2.7
ΔE_{DFT}/cm^{-1}	509.7	535.3	591.6

[a] See footnote of Table S1.

[b] Absolute energy is -802.1296231 E_h.

Table S6. Experimental rotational parameters obtained for the observed ^{13}C and D isotopologues of conformer I of salicylic acid. The D species were also fit with the transitions of the millimetre-wave previous study [11]. The centrifugal distortion constants for all the isotopologues were fixed to the parent values. See Figure 3 or Table S9 for the atom labelling.

Fitted Param.[a]	$^{13}C_1$	$^{13}C_2$	$^{13}C_3$	$^{13}C_4$	$^{13}C_5$
A/MHz	2339.6062(13)[b]	2330.4567(20)	2330.9608(16)	2339.51690(98)	2317.03428(97)
B/MHz	1223.74657(95)	1222.93965(80)	1212.7043(10)	1203.85694(74)	1212.8771(11)
C/MHz	803.81184(76)	802.37992(76)	798.02404(70)	795.17425(46)	796.45755(66)
P_{cc}/uÅ^2	0.12957(52)	0.12884(53)	0.13036(52)	0.13043(36)	0.12946(50)
N	11	7	10	10	13
σ/kHz	9.5	7.4	8.6	5.5	8.1
	$^{13}C_6$	$^{13}C_{11}$			
A/MHz	2317.03232(94)	2339.7700(13)			
B/MHz	1222.93849(56)	1215.9562(12)			
C/MHz	800.78462(60)	800.46268(82)			
P_{cc}/uÅ^2	0.12987(38)	0.12963(59)			
N	13	10			
σ/kHz	6.9	8.4			
	D_{15}	D_{16}	D_{15}-D_{16}		
A/MHz	2323.77041(21)	2297.80023(28)	2280.69331(29)		
B/MHz	1194.46860(18)	1220.49531(35)	1191.70809(50)		
C/MHz	798.26545(21)	797.43514(34)	783.04691(44)		
P_{cc}/uÅ^2	0.13307(13)	0.13085(21)	0.13446(28)		
N	53[c]	52[c]	43[c]		
σ/kHz	15.4	21.5	19.4		

[a] A, B and C are the rotational constants. P_{cc} is the planar moment of inertia, derived from $P_{cc}=(I_a+I_b-I_c)/2$. Δ_J, Δ_{JK}, Δ_K, δ_J and δ_K are the quartic centrifugal distortion constants. N is the number of rotational transitions fitted. σ is the rms deviations of the fit.

[b] Standard error is given in parentheses in units of the last digit.

[c] Fit done with the transition measured in the previous work [11].

Table S7. Experimental rotational parameters obtained for the observed D isotopologues of conformer SA-w (I-w-a) of salicylic acid – water cluster. The centrifugal distortion constants for all the isotopologues were fixed to the parent values. See Figure S3 or Table S12 for the atom labelling.

**Fitted Param.**[a]	**D_{15}**	**D_{16}**	**D_{18}**	**D_{19}**
A/MHz	2303.2538(24)[b]	2270.4682(19)	2311.152(15)	2311.8536(35)
B/MHz	638.94582(43)	644.94882(32)	634.33584(77)	625.31918(61)
C/MHz	500.70308(30)	502.79168(22)	498.25634(52)	492.99050(33)
P_{cc}/uÅ^2	0.51920(68)	0.51881(51)	0.5401(17)	0.83388(90)
N	14	13	10	11
σ/kHz	7.2	5.5	7.7	8.9

[a] A, B and C are the rotational constants. P_{cc} is the planar moment of inertia, derived from $P_{cc}=(I_a+I_b-I_c)/2$. N is the number of rotational transitions fitted. σ is the rms deviations of the fit.

[b] Standard error is given in parentheses in units of the last digits.

Table S8. r_s and r_m geometry parameters (lengths in Å and angles in degree) experimentally determined and their comparison with the r_e structure predicted at B3LYP-D3/6-311++G(d,p) level of theory and with the r_a structure obtained by gas-phase electron diffraction [12] for the conformer I of salicylic acid. For the r_m structure the $r_m^{(1L)}$ definition has been chosen. This definition employs C_α coefficients to reproduce the vibration-rotation term in order to obtain the mass-dependant moments of inertia from the effective ones. The different C_α constants were estimated to have similar values so in the final fits these were assumed to be equal. The L refers to the parameter δ_H for all r_m-r(C-H) bonds, which in this case was fixed to 0.01 Å [60]. See Figure 3 or Table S9 for atom labelling.

Parameter	r_s	$r_m^{(1L)}$	r_a [a]	r_e
$r(C_1-C_2)$	-	1.4091(51)	1.419(3)	1.417
$r(C_2-C_3)$	1.4114(31)[b]	1.4064(67)	1.407(6)	1.402
$r(C_3-C_4)$	1.3813(51)	1.3809(96)	1.395(4)	1.384
$r(C_4-C_5)$	1.4083(50)	1.4120(87)	1.413(6)	1.402
$r(C_5-C_6)$	1.3920(30)	1.3841(73)	1.396(4)	1.383
$r(C_1-C_6)$	-	1.4031(79) [c]	1.410(6)	1.408
$r(C_1-C_{11})$	-	1.4682(83)	1.465(8)	1.465
$r(C_2-O_{12})$	-	1.342(13)	1.333(7)	1.344
$r(C_{11}-O_{13})$	-	1.3496(42) [c]	1.339(7)	1.350
$r(C_{11}-O_{14})$	-	1.2258(42) [c]	1.228(6)	1.226
$r(O_{12}-H_{16})$	-	0.990(14)	1.051(20)	0.980
$r(O_{13}-H_{15})$	-	0.9685(19) [c]	1.063(20)	0.968
$r(O_{14}\cdots H_{16})$	-	1.7569(40) [c]	1.727(14)	1.775
$\angle(C_1-C_2-C_3)$	-	119.2(5) [c]	120.0(3)	119.2
$\angle(C_2-C_3-C_4)$	120.1(2)	120.1(3)	120.8(3)	120.2
$\angle(C_3-C_4-C_5)$	120.8(1)	120.9(1)	118.5(3)	121.0
$\angle(C_4-C_5-C_6)$	119.2(1)	119.1(3)	122.0(3)	119.3
$\angle(C_1-C_6-C_5)$	-	120.7(5) [c]	119.1(3)	120.9
$\angle(C_2-C_1-C_6)$	-	119.9(7) [c]	119.6(3)	119.3
$\angle(C_2-C_1-C_{11})$	-	118.4(5) [c]	-	119.0
$\angle(C_6-C_1-C_{11})$	-	121.7(5) [c]	-	121.7
$\angle(C_1-C_2-O_{12})$	-	123.5(8) [c]	-	123.3
$\angle(C_3-C_2-O_{12})$		117.2(6)	-	117.5
$\angle(C_1-C_{11}-O_{13})$	-	113.9(3)	-	114.7
$\angle(C_1-C_{11}-O_{14})$	-	125.3(4) [c]	-	124.5
$\angle(C_2-O_{12}-H_{16})$	-	107.6(6)	-	108.2
$\angle(C_{11}-O_{13}-H_{15})$	-	105.4(2)	-	107.1
$\angle(O_{12}-H_{16}-O_{14})$	-	145.4(3) [c]	-	144.5
C_α[d] / $u^{1/2} \cdot$ Å	-	0.01742(35)	-	-
δ_H / $u^{1/2} \cdot$ Å	-	[0.010][e]	-	-
σ_{fit}[f] / u · Å^2	-	0.009792	-	-

[a] The r_a structure has been taken from the reference 12. [b] Standard error is given in parenthesis in units of the last digit. [c] Derived parameters. [d] Fitted using the assumption $C_\alpha = C_a = C_b = C_c$. [e] Fixed values for H_{15} and H_{16}. [f] Standard deviation of the fit of all the rotational constants.

Table S9. Principal inertial axis coordinates for the atoms of conformer I of salicylic acid. The table compares the theoretical coordinates (r_e) calculated at the B3LYP-D3/6-311++G(d,p) level of theory with the experimental r_s and r_m coordinates. The definition $r_m^{(1L)}$ was chosen for the r_m determination. [60]. The hydrogen H_{15} and H_{16} coordinates were also compared with the obtained r_s coordinates in the previous study [11].

atom	method	*a*	*b*	*c*
**C_1**	r_e	0.1737	0.2305	0.000
	r_m	0.1675(82)[a]	0.2623(59)	0.000
	r_s	[0.000][b]	0.2446(34)	0.000
**C_2**	r_e	-0.5442	-0.9618	0.000
	r_m	-0.5362(63)	-0.9585(42)	0.000
	r_s	-0.5292(30)	-0.9552(16)	0.000
**C_3**	r_e	-1.9455	-0.9330	0.000
	r_m	-1.9425(20)	-0.9411(48)	0.000
	r_s	-1.94056(78)	-0.9384(16)	0.000
**C_4**	r_e	-2.6183	0.2770	0.000
	r_m	-2.6201(14)	0.2622(89)	0.000
	r_s	-2.61820(58)	0.2652(57)	0.000
**C_5**	r_e	-1.9182	1.4923	0.000
	r_m	-1.9210(21)	1.4890(29)	0.000
	r_s	-1.91901(78)	1.4877(10)	0.000
**C_6**	r_e	-0.5356	1.4766	0.000
	r_m	-0.5370(70)	1.4758(30)	0.000
	r_s	-0.5271(29)	1.4749(10)	0.000
**H_7**	r_e	-2.4743	-1.8779	0.000
	r_m	-2.4635(49)	-1.8904(64)	0.000
	r_s	-	-	-
**H_8**	r_e	-3.7027	0.2806	0.000
	r_m	-3.7046(14)	0.260(13)	0.000
	r_s	-	-	-
**H_9**	r_e	-2.4542	2.4332	0.000
	r_m	-2.4614(41)	2.4274(32)	0.000
	r_s	-	-	-
**H_{10}**	r_e	0.0249	2.4023	0.000
	r_m	0.0218(66)	2.4025(41)	0.000
	r_s	-	-	-
**C_{11}**	r_e	1.6381	0.2238	0.000
	r_m	1.6351(14)	0.2225(36)	0.000
	r_s	1.63196(93)	0.2128(71)	0.000
**O_{12}**	r_e	0.0514	-2.1663	0.000
	r_m	0.063(14)	-2.1592(40)	0.000
	r_s	-	-	-

[a] Standard error is given in parentheses in units of the last digits.

[b] r_s parameters in square brackets are fixed to zero owing to the Kraitchman equations give imaginary values.

Table S9 (Continued).

atom	method	*a*	*b*	*c*
**O_{13}**	r_e	2.2323	1.4356	0.000
	r_m	2.2153(15)[a]	1.4411(28)	0.000
	r_s	-	-	-
**O_{14}**	r_e	2.3070	-0.8034	0.000
	r_m	2.3157(33)	-0.7969(26)	0.000
	r_s	-	-	-
**H_{15}**	r_e	3.1887	1.2831	0.000
	r_m	3.1689(11)	1.2722(28)	0.000
	r_s	3.17210(86)	1.2696(12)	0.000
	r_s [c]	3.173(1)	1.270(1)	0.000
**H_{16}**	r_e	1.0221	-2.0284	0.000
	r_m	1.0412(33)	-2.0068(17)	0.000
	r_s	1.0414(11)	-2.0064(17)	0.000
	r_s [c]	1.045(5)	-2.004(1)	0.000

[a] Standard error is given in parentheses in units of the last digit.
[c] r_s coordinates taken from reference 11.

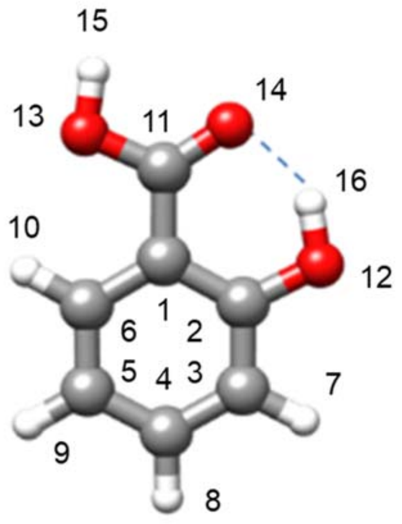

Table S10. Predicted stabilizing delocalization energies from the NBO calculations done at B3LYP-D3/6-311++G(d,p) level of theory for the observed I, I-w-a, I-w$_2$-a, I-w$_3$-a and I-w$_4$-a species of salicylic acid and its hydrates. The delocalization energies in the first case are the donor-acceptor conjugative stabilization energies which informs about the charge transfer between $\pi \leftarrow \pi^*$ orbitals. In the second case they are the electron delocalization energies from the lone pair n_O of the oxygen atoms to the σ^* orbital of the hydrogen atoms, which characterize the hydrogen bonds.

π	π*	E / kcal·mol^{-1}				
		**I**	**I-w-a**	**I-w$_2$-a**	**I-w$_3$-a**	**I-w$_4$-a**
C_1-C_2	C_3-C_4	13.32	13.45	13.52	14.20	14.21
	C_5-C_6	22.88	22.87	22.85	24.01	23.92
	C_{11}-O_{14}	29.66	30.88	31.20	31.07	32.13
C_3-C_4	C_1-C_2	24.44	24.31	24.27	26.02	26.04
	C_5-C_6	15.55	15.47	15.47	15.55	15.49
C_5-C_6	C_1-C_2	15.00	15.00	14.01	16.12	16.15
	C_3-C_4	22.61	22.77	22.82	22.93	23.04
C_{11}-O_{14}	C_1-C_2	2.84	2.67	2.55	3.20	3.11
**n_O**	**σ***					
O_{12}	C_1-C_2	37.21	36.93	36.64	41.99	41.79
O_{13}	C_{11}-O_{14}	45.23	53.96	59.03	62.85	68.7
O_{14}	C_1-C_{11}	13.13	12.43	12.89	13.42	13.93
	C_{11}-O_{13}	30.40	26.45	24.86	25.32	23.47
	O_{12}-H_{16}	16.02	17.15	17.21	18.32	18.11
O_{17}	O_{13}-H_{15}	-	17.59	29.75	30.46	42.55
O_{14}	O_{17}-H_{18}	-	3.08	-	-	-
	O_{20}-H_{21}	-	-	4.39	2.73	8.41
O_{20}	O_{17}-H_{18}	-	-	16.96	-	0.19
	O_{23}-H_{24}	-	-	-	21.33	-
	O_{26}-H_{27}	-	-	-	-	12.89
O_{23}	O_{17}-H_{18}	-		-	18.25	-
	O_{17}-H_{19}	-	-	-	-	16.19
O_{26}	O_{23}-H_{24}	-	-	-	-	15.04

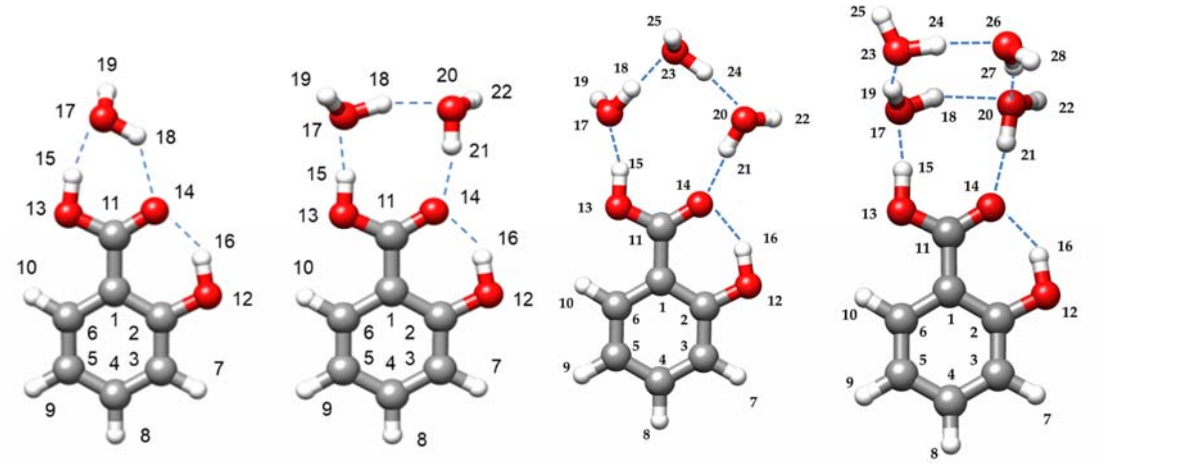

Table S11. Predicted bond orders from the NBO calculations done at B3LYP-D3/6-311++G(d,p) level of theory for the observed I, I-w-a, I-w2-a, I-w3-a and I-w4-a species of salicylic acid and its hydrates.

		I	I-w-a	I-w2-a	I-w3-a	I-w4-a
C_1	C_2	1.3789	1.3869	1.3882	1.3209	1.3361
	C_6	1.4011	1.4034	1.3991	1.4120	1.4261
	C_{11}	1.0299	1.0287	1.0266	1.0313	1.0369
C_2	C_3	1.3993	1.4044	1.4025	1.4051	1.3906
	O_{12}	1.0941	1.0931	1.0917	1.1207	1.1283
C_3	C_4	1.4869	1.4958	1.4859	1.5712	1.5537
C_4	C_5	1.4355	1.4379	1.4426	1.4301	1.4370
C_5	C_6	1.4922	1.5036	1.5002	1.5431	1.5400
C_{11}	O_{13}	1.2120	1.3003	1.3314	1.1943	1.3029
	O_{14}	1.7204	1.6592	1.6285	1.5783	1.4917
O_{12}	H_{16}	0.9728	0.9720	0.9719	0.8902	0.8978
O_{13}	H_{15}	0.9886	0.9699	0.9580	0.8283	0.7508
O_{17}	H_{18}	-	1.0000	0.9802	0.8637	0.9544
	H_{19}	-	0.9919	1.0000	1.0000	0.8949
O_{20}	H_{21}	-	-	0.9854	0.9364	0.9176
	H_{22}	-	-	1.0000	1.0000	1.0000
O_{23}	H_{24}	-	-	-	0.8852	0.9039
	H_{25}	-	-	-	1.0000	1.0000
O_{26}	H_{27}	-	-	-	-	0.9186
	H_{28}	-	-	-	-	1.0000

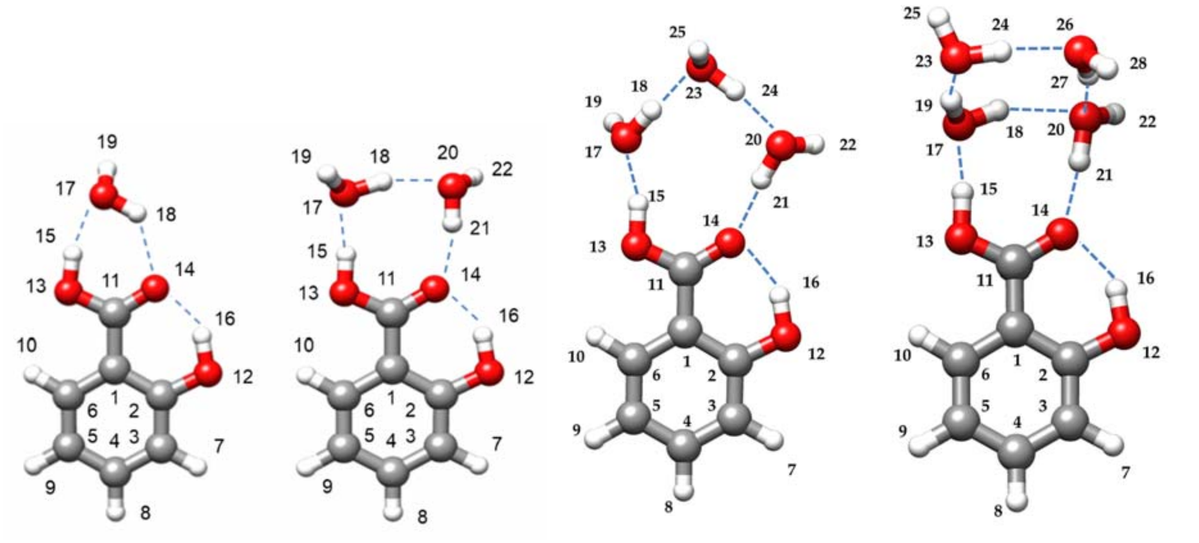

Table S12. $r_{s,}$ and r_0 geometry parameters (lengths in Å and angles in degree) experimentally determined and their comparison with the r_e structure predicted at B3LYP-D3/6-311++G(d,p) level of theory for the conformer I-w-a of salicylic acid monohydrated cluster.

**Parameter**	r_s	r_0	r_e
r(H$_{15}$-H$_{18}$)	1.9287(44)[a]	1.9223(54)[b]	1.993
r(C$_{11}$-O$_{13}$)	-	1.3496(10)[b]	1.330
r(C$_{11}$-O$_{14}$)	-	1.22576(98)[b]	1.239
r(C$_{12}$-O$_{16}$)	-	1.0490(80)	0.981
r(O$_{13}$-H$_{15}$)	-	1.0065(13)	0.988
r(O$_{17}$-H$_{18}$)	-	0.9650(42)[b]	0.972
r(O$_{14}$···H$_{16}$)	-	1.7019(60)[b]	1.754
r(H$_{15}$···O$_{17}$)	-	1.7531(24)	1.764
r(O$_{14}$···H$_{18}$)	-	1.9819(25)	2.031
∠(C$_{11}$-O$_{13}$-H$_{15}$)	-	108.5(1)	108.9
∠(C$_{2}$-O$_{12}$-H$_{16}$)	-	107.0(1)	107.8
∠(O$_{13}$-H$_{15}$···O$_{17}$)	-	160.6(2)	158.9
∠(C$_{11}$-O$_{14}$···H$_{!8}$)	-	109.2(2)	109.5
∠(O$_{14}$····H$_{18}$-O$_{17}$)	-	136.7(4)[b]	132.1
∠(O$_{13}$-H$_{15}$···O$_{17}$-H$_{19}$)	-	119.4(8)	117.9
∠(C$_{11}$-O$_{13}$-H$_{15}$···O$_{17}$)		-13.8(5)	-4.5
∠(C$_{1}$-C$_{11}$-O$_{14}$···H$_{18}$)		173.9(5)	179.8
σ_{fit}^{c} / u · Å^2	-	0.005575	-

[a] Standard error is given in parenthesis in units of the last digit.
[b] Derived parameters.
[c] Standard deviation of the fit of all the rotational constants.

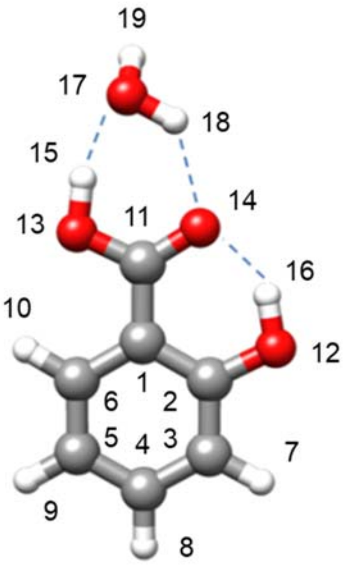

Table S13. Principal inertial axis coordinates for the selected atoms of conformer I-w-a of salicylic acid monohydrated cluster. The table compares the theoretical coordinates calculated at the B3LYP-D3/6-311++G(d,p) level of theory with the experimental r_s and r_0 coordinates.

atom	method	*a*	*b*	*c*
**H15**	r_e	2.7275	1.1018	-0.0056
	r_s	2.73986(55)[a]	1.0767(14)	-0.046(33)
	r_0	2.7404(11)	1.0744(27)	-0.0406(16)
**H16**	r_e	0.3529	-2.0915	0.0026
	r_s	0.3976(38)	-2.07723(73)	[0.000][b]
	r_0	0.4015(77)	-2.0761(14)	0.01323(39)
**H18**	r_e	3.7000	-0.6373	0.0035
	r_s	3.63884(43)	-0.6182(26)	0.152(11)
	r_0	3.63926(90)	-0.6140(50)	0.151(19)
**H19**	r_e	4.9103	0.1122	-0.6267
	r_s	4.94329(31)	0.117(13)	-0.5712(27)
	r_0	4.94376(64)	0.045(12)	-0.5742(46)

[a] Standard error is given in parentheses in units of the last digit.

[b] r_s parameters in square brackets are fixed to zero owing to the Kraitchman equations give imaginary values.

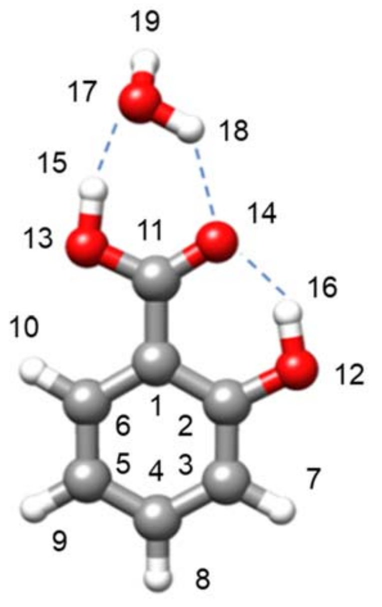

Table S14. r_e geometrical parameters (lengths in Å and angles in degree) predicted at B3LYP-D3/6-311++G(d,p) level of theory for the conformer I-w$_2$-a of salicylic acid dihydrated cluster.

Parameter	r_e
r(O_{12}-H_{16})	0.980
r(O_{14}···H_{16})	1.749
r(C_{11}-O_{13})	1.317
r(C_{11}-O_{14})	1.243
r(O_{13}-H_{15})	1.002
r(O_{17}-H_{18})	0.984
r(O_{20}-H_{21})	0.979
r(H_{15}···O_{17})	1.647
r(O_{14}···H_{21})	1.806
r(O_{20}···H_{18})	1.749
∠(O_{13}-H_{15}···O_{17})	176.3
∠(O_{14}···H_{21}-O_{20})	166.0
∠(O_{17}-H_{18}···O_{20})	157.7

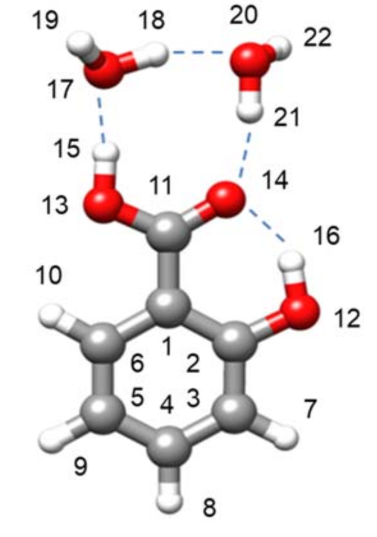

Table S15. r_e geometrical parameters (lengths in Å and angles in degree) predicted at B3LYP-D3/6-311++G(d,p) level of theory for the conformer I-w$_3$-a of salicylic acid trihydrated cluster.

Parameter	r_e
r(O_{12}-H_{16})	0.981
r(O_{14}···H_{16})	1.748
r(C_{11}-O_{13})	1.314
r(C_{11}-O_{14})	1.243
r(O_{13}-H_{15})	1.004
r(O_{17}-H_{18})	0.988
r(O_{20}-H_{21})	0.978
r(O_{23}-H_{24})	0.984
r(H_{15}···O_{17})	1.627
r(O_{14}···H_{21})	1.787
r(O_{20}···H_{24})	1.734
r(O_{23}···H_{18})	1.707
∠(O_{13}-H_{15}···O_{17})	167.4
∠(O_{14}···H_{21}-O_{20})	175.7
∠(O_{17}-H_{18}···O_{23})	173.3
∠(O_{23}-H_{24}···O_{20})	165.6
∠(C_1-C_{11}-O_{13}-H_{15})	-177.4
∠(C_1-C_{11}-O_{13}···O_{17})	-175.7
∠(C_1-C_{11}-O_{13}···O_{23})	170.5
∠(C_1-C_{11}-O_{14}···O_{20})	-153.7
∠(C_1-C_{11}-O_{14}···O_{23})	-169.1

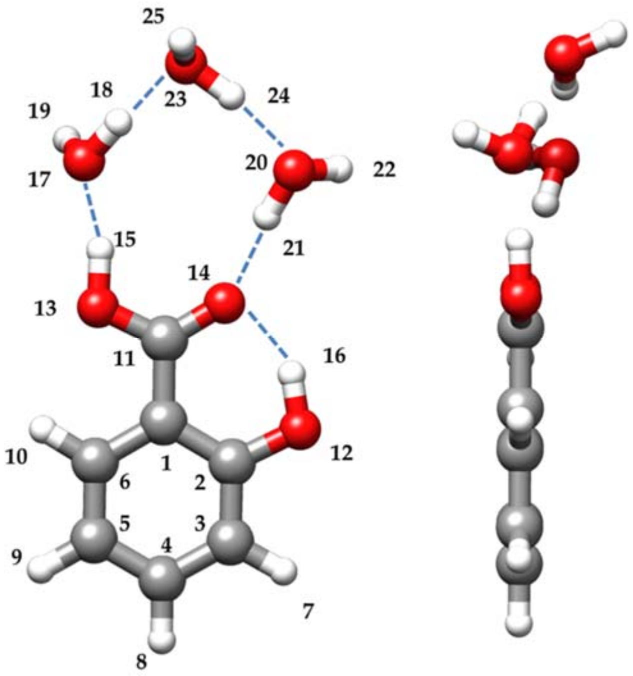

Table S16. r_e geometrical parameters (lengths in Å and angles in degree) predicted at B3LYP-D3/6-311++G(d,p) level of theory for the conformer I-w$_4$-a of salicylic acid tetrahydrated cluster.

Parameter	r_e
r(O$_{12}$-H$_{16}$)	0.981
r(O$_{14}$···H$_{16}$)	1.742
r(C$_{11}$-O$_{13}$)	1.307
r(C$_{11}$-O$_{14}$)	1.250
r(O$_{13}$-H$_{15}$)	1.020
r(O$_{17}$-H$_{18}$)	0.970
r(O$_{17}$-H$_{19}$)	0.983
r(O$_{20}$-H$_{21}$)	0.987
r(O$_{23}$-H$_{24}$)	0.981
r(O$_{26}$-H$_{27}$)	0.978
r(H$_{15}$···O$_{17}$)	1.557
r(O$_{14}$···H$_{21}$)	1.710
r(O$_{20}$···H$_{18}$)	1.972
r(O$_{20}$···H$_{27}$)	1.817
r(O$_{23}$···H$_{19}$)	1.779
r(O$_{26}$···H$_{24}$)	1.789
∠(O$_{13}$-H$_{15}$···O$_{17}$)	175.9
∠(O$_{14}$···H$_{21}$-O$_{20}$)	174.7
∠(O$_{17}$-H$_{18}$···O$_{20}$)	146.9
∠(O$_{17}$-H$_{19}$···O$_{23}$)	165.1
∠(O$_{23}$-H$_{24}$···O$_{26}$)	164.8
∠(O$_{26}$-H$_{27}$···O$_{20}$)	167.9
∠(C$_1$-C$_{11}$-O$_{13}$···O$_{17}$)	179.6
∠(C$_1$-C$_{11}$-O$_{14}$···O$_{20}$)	179.0
∠(O$_{13}$···O$_{17}$···O$_{23}$)	114.6
∠(O$_{14}$···O$_{20}$···O$_{26}$)	110.8
∠(O$_{13}$···O$_{17}$···O$_{20}$···O$_{26}$)	-109.6
∠(O$_{13}$···O$_{17}$···O$_{20}$···O$_{26}$)	113.8
∠(C$_{11}$-O$_{13}$···O$_{17}$···O$_{23}$)	-92.6
∠(C$_{11}$-O$_{14}$···O$_{20}$···O$_{26}$)	87.4
∠(O$_{17}$···O$_{20}$···O$_{26}$···O$_{23}$)	-0.9

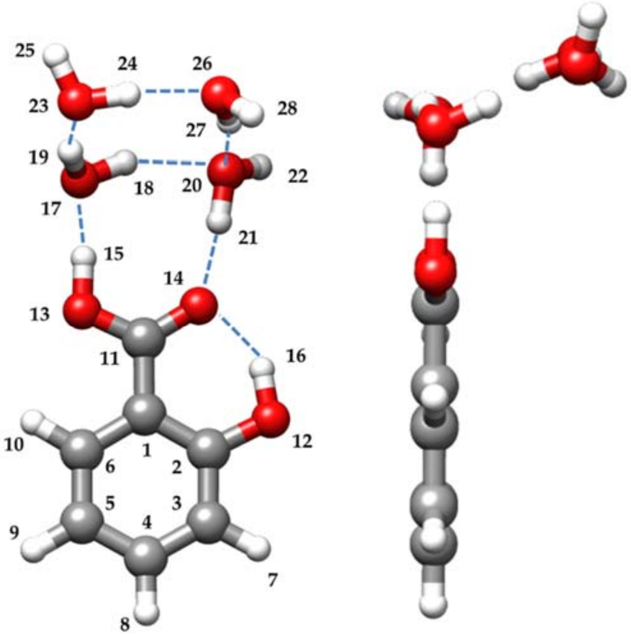

Table S17. Observed rotational transitions and residuals (all the values in MHz) for conformer I of the salicylic acid in the ground vibrational state. The lines with frequencies above 8 GHz were measured in the MB-FTMW spectrometer as a thermal recombination product in the spectroscopic analysis of *o*-anisic acid [36].

J′	K_{-1}′	K_{+1}′	J′′	K_{-1}′′	K_{+1}′′	Obs.	Obs.-Cal.
1	1	1	0	0	0	3144.1569	0.0084
2	0	2	1	1	1	2840.9720	-0.0044
2	1	2	1	0	1	4751.9445	0.0007
2	2	1	1	1	0	7824.6411	-0.0060
2	1	1	2	0	2	2054.0953	-0.0037
2	2	0	2	1	1	3447.3062	-0.0122
2	2	1	2	1	2	4609.0603	0.0043
3	1	3	2	1	2	5397.7466	-0.0146
3	2	1	2	2	0	6443.8813	0.0179
3	0	3	2	1	2	4927.6352	-0.0115
3	1	3	2	0	2	6192.2528	0.0055
3	1	2	2	2	1	3293.1922	-0.0033
3	2	2	2	1	1	9432.4424	0.0014
3	1	2	3	0	3	2974.6056	0.0007
3	2	1	3	1	2	3248.5485	0.0052
3	2	2	3	1	3	5294.2991	0.0064
3	3	0	3	2	1	6243.5029	0.0014
3	3	1	3	2	2	6687.5098	0.0020
4	0	4	3	0	3	7344.3581	0.0050
4	0	4	3	1	3	6874.2407	0.0021
4	1	4	3	0	3	7581.6410	-0.0111
4	1	3	3	2	2	5916.1901	-0.0027
4	1	3	4	0	4	4336.2379	-0.0090
4	2	2	4	1	3	3340.5304	-0.0006
4	2	3	4	1	4	6216.2529	-0.0077
4	3	1	4	2	2	5800.5691	-0.0151
4	3	2	4	2	3	6924.0407	-0.0047
4	4	1	4	3	2	9147.2357	-0.0015
5	0	5	4	1	4	8675.8944	0.0004
5	1	5	4	0	4	9020.2548	0.0005
5	1	4	4	2	3	8491.9491	-0.0002
5	2	3	4	3	2	5431.8666	0.0022
5	1	4	5	0	5	6032.3278	0.0116
5	2	3	5	1	4	3863.9545	-0.0058
5	2	4	5	1	5	7355.1534	-0.0039
5	3	2	5	2	3	5290.7705	-0.0007

J′	K_{-1}′	K_{+1}′	J′′	K_{-1}′′	K_{+1}′′	Obs.	Obs.-Cal.
1	1	1	0	0	0	3144.1569	0.0084
5	3	3	5	2	4	7352.2379	0.0009
5	4	2	5	3	2	8795.5073	0.0002
5	4	3	5	3	3	9151.9816	0.0001
6	2	6	5	3	3	8441.1694	0.0001
6	2	5	5	3	2	4016.5305	-0.0099
6	3	5	5	4	2	4236.6208	0.0013
6	2	3	6	1	5	4899.8952	-0.0094
6	2	4	6	1	6	8672.1102	0.0001
6	3	6	6	2	4	4947.4359	0.0043
6	4	5	6	3	3	8321.1307	-0.0003
6	4	4	6	3	4	9234.4157	0.0005
7	2	5	6	3	3	4494.8206	0.0025
7	3	6	6	4	3	7226.8784	0.0017
7	3	5	6	4	2	5078.3827	-0.0022
7	1	7	7	0	7	9690.3441	-0.0013
7	2	3	7	1	6	6427.5530	0.0017
7	3	1	7	2	5	4993.9969	0.0087
7	3	2	7	2	6	8904.6994	0.0014
7	4	4	7	3	4	7651.2910	0.0085
7	4	5	7	3	5	9456.0961	0.0010
8	2	3	8	1	7	8289.4650	0.0010
8	3	4	8	2	6	5576.9324	-0.0152
8	4	5	8	3	5	6963.7282	0.0040
8	4	3	8	3	6	9873.7942	0.0011
9	3	2	9	2	7	6752.9531	-0.0135
9	4	3	9	3	6	6524.7030	-0.0159
10	3	6	10	2	8	8454.1179	0.0003
10	5	4	10	4	6	9351.3204	-0.0003
5	3	5	5	2	4	7352.2379	0.0009

Table S18. Observed rotational transitions and residuals (all the values in MHz) for the ^{13}C isotopomers of conformer I of salicylic acid in the ground vibrational state.

$^{13}C_1$

J′	K_{-1}′	K_{+1}′	J′′	K_{-1}′′	K_{+1}′′	Obs.	Obs.-Cal.
2	1	2	1	0	1	4751.0232	-0.0181
3	1	3	2	0	2	6191.1651	-0.0094
3	3	0	3	2	1	6240.3156	0.0098
4	0	4	3	1	3	6873.9352	0.0062
4	1	4	3	0	3	7580.4789	0.0016
4	1	3	3	2	2	5917.5879	0.0081
4	1	3	4	0	4	4336.4057	0.0054
4	2	3	4	1	4	6214.8713	-0.0136
4	3	1	4	2	2	5797.2780	0.0111
5	3	2	5	2	3	5287.5748	-0.0043
6	3	3	6	2	4	4944.7776	-0.0003

$^{13}C_2$

J′	K_{-1}′	K_{+1}′	J′′	K_{-1}′′	K_{+1}′′	Obs.	Obs.-Cal.
2	1	2	1	0	1	4737.6000	0.0039
3	0	3	2	1	2	4926.2982	-0.0030
3	1	3	2	0	2	6174.9609	0.0000
4	0	4	3	1	3	6867.0631	0.0116
4	1	3	3	2	2	5932.5785	-0.0113
4	3	2	4	2	3	6883.4627	-0.0077
5	1	4	5	0	5	6033.8355	0.0061

$^{13}C_3$

J′	K_{-1}′	K_{+1}′	J′′	K_{-1}′′	K_{+1}′′	Obs.	Obs.-Cal.
2	1	2	1	0	1	4725.0354	0.0028
3	0	3	2	1	2	4880.0778	-0.0088
3	1	3	2	0	2	6155.1422	0.0087
3	2	2	3	1	3	5274.9843	-0.0171
4	0	4	3	1	3	6815.5527	-0.0042
4	1	4	3	0	3	7533.4870	0.0028
4	1	3	4	0	4	4292.5050	-0.0042
4	2	3	4	1	4	6185.0092	-0.0004
4	3	2	4	2	3	6913.4251	-0.0023
5	2	4	5	1	5	7309.8770	0.0150

$^{13}C_4$

J′	K_{-1}′	K_{+1}′	J′′	K_{-1}′′	K_{+1}′′	Obs.	Obs.-Cal.
2	1	2	1	0	1	4725.0354	-0.0038
2	1	1	2	0	2	2044.9233	-0.0071
3	0	3	2	1	2	4837.5965	-0.0072
3	1	3	2	0	2	6150.7572	0.0072
4	0	4	3	1	3	6772.7007	0.0054
4	1	4	3	0	3	7522.3372	-0.0035
4	1	3	4	0	4	4251.8141	0.0036
4	2	3	4	1	4	6193.9011	-0.0048
4	3	2	4	2	3	6975.2528	-0.0033
5	3	3	5	2	4	7380.7390	0.0068

$^{13}C_5$

J′	K_{-1}′	K_{+1}′	J′′	K_{-1}′′	K_{+1}′′	Obs.	Obs.-Cal.
2	2	1	1	1	0	7747.5599	0.0024
3	0	3	2	1	2	4884.3544	-0.0095
3	1	3	2	0	2	6133.3180	0.0039
3	2	1	3	1	2	3213.5115	-0.0041
3	2	2	3	1	3	5241.4663	0.0190
3	3	0	3	2	1	6175.7001	-0.0021
3	3	1	3	2	2	6616.9130	-0.0046
4	0	4	3	1	3	6812.3164	0.0038
4	1	4	3	0	3	7510.0423	-0.0008
4	2	3	4	1	4	6155.9342	-0.0149
4	3	2	4	2	3	6851.9933	0.0056
5	2	3	5	1	4	3827.6305	0.0070
5	3	3	5	2	4	7277.4019	-0.0044

$^{13}C_6$

J′	K_{-1}′	K_{+1}′	J′′	K_{-1}′′	K_{+1}′′	Obs.	Obs.-Cal.
1	1	1	0	0	0	3117.8024	-0.0144
2	1	2	1	0	1	4719.3813	-0.0044
2	2	1	1	1	0	7751.8921	0.0134
3	0	3	2	1	2	4929.4646	0.0081
3	1	3	2	0	2	6153.5491	0.0033
3	2	1	3	1	2	3191.0841	-0.0058
4	0	4	3	1	3	6862.7556	-0.0040
4	1	3	4	0	4	4341.8484	0.0007
4	2	2	4	1	3	3298.0640	0.0045
4	3	1	4	2	2	5681.1387	-0.0058
4	3	2	4	2	3	6824.8598	-0.0011
5	2	3	4	3	2	5546.8172	-0.0044
5	3	3	5	2	4	7262.2342	-0.0008

$^{13}C_{11}$

J′	K_{-1}′	K_{+1}′	J′′	K_{-1}′′	K_{+1}′′	Obs.	Obs.-Cal.
2	1	2	1	0	1	4741.1617	0.0041
2	2	1	1	1	0	7819.7501	-0.0196
3	0	3	2	1	2	4892.5132	0.0069
3	1	3	2	0	2	6175.7001	0.0015
3	2	1	3	1	2	3264.9584	0.0017
4	0	4	3	1	3	6834.5550	0.0014
4	2	3	4	1	4	6206.9918	0.0016
4	3	2	4	2	3	6943.1865	-0.0005
5	2	4	5	1	5	7334.0486	-0.0055
5	3	3	5	2	4	7362.4729	0.0149

Table S19. Observed rotational transitions and residuals (all the values in MHz) for the D isotopomer of conformer I of salicylic acid in the ground vibrational state.

D_{15}

J′	K_{-1}′	K_{+1}′	J′′	K_{-1}′′	K_{+1}′′	Obs.	Obs.-Cal.
3	0	3	2	1	2	4799.1786	-0.0144
3	1	3	2	0	2	6106.7554	-0.0029
3	2	1	3	1	2	3272.8064	0.0158
3	2	2	3	1	3	5262.9475	-0.0111
4	0	4	3	1	3	6720.5355	0.0053
4	1	4	3	0	3	7467.9881	0.0000
4	1	3	3	2	2	5675.3085	0.0099
4	1	3	4	0	4	4217.7278	0.0006
4	3	2	4	2	3	6931.8336	-0.0065
4	2	3	4	1	4	6150.8743	0.0004
4	3	1	4	2	2	5872.8458	-0.0062
5	1	4	5	0	5	5860.8630	0.0000
5	2	3	5	1	4	3806.4400	0.0064
6	1	5	6	0	6	7659.3144	0.0024
6	2	4	6	1	5	4766.3271	-0.0011
6	3	3	6	2	4	5000.6281	0.0026
7	3	4	6	4	3	6716.5451	0.0000
7	2	5	7	1	6	6210.2823	0.0003
7	3	4	7	2	5	4985.7092	0.0020
7	4	3	7	3	4	7792.6605	0.0051
8	4	5	7	5	2	4665.9047	-0.0011
8	3	5	8	2	6	5473.3292	-0.0036
8	4	4	8	3	5	7103.0519	0.0076
9	3	6	9	2	7	6531.3548	0.0018
9	4	5	9	3	6	6611.2054	-0.0083
10	4	6	10	3	7	6552.8229	-0.0005
11	4	7	11	3	8	7080.7922	-0.0009

D_{16}

J′	K_{-1}′	K_{+1}′	J′′	K_{-1}′′	K_{+1}′′	Obs.	Obs.-Cal.
4	1	1	2	0	2	2025.5524	-0.0010
4	0	3	2	1	2	4922.8451	-0.0155
2	1	3	2	0	2	6117.9076	0.0058
3	1	2	3	0	3	2961.0767	0.0130
3	2	1	3	1	2	3147.6445	0.0035
3	2	2	3	1	3	5193.3566	0.0009
3	3	0	3	2	1	6041.1232	0.0046
3	3	1	3	2	2	6501.8394	-0.0020
3	0	4	3	1	3	6844.1195	0.0083
3	1	4	3	0	3	7499.0788	0.0007
4	1	3	3	2	2	5989.8802	-0.0017
4	1	3	4	0	4	4339.1256	-0.0007
4	2	3	4	1	4	6124.0846	0.0115
4	3	1	4	2	2	5591.8242	0.0077
4	3	2	4	2	3	6747.6209	0.0071
4	1	4	5	0	5	6040.6141	0.0014
4	2	3	5	1	4	3825.6747	-0.0118
5	2	4	5	1	5	7271.3925	0.0044

D_{16}

J′	K_{-1}′	K_{+1}′	J′′	K_{-1}′′	K_{+1}′′	Obs.	Obs.-Cal.
5	2	4	6	1	5	4906.7594	-0.0086
5	3	3	6	2	4	4782.5530	-0.0005
6	3	4	6	2	5	7866.5361	0.0035
6	2	5	7	1	6	6471.9922	-0.0019
6	3	4	7	2	5	4887.8558	0.0000
7	3	5	8	2	6	5546.7901	0.0046
7	4	4	8	3	5	6671.7283	-0.0069
8	3	6	9	2	7	6802.2215	0.0030
8	4	5	9	3	6	6299.5059	-0.0133
9	4	6	10	3	7	6447.4680	-0.0084
9	4	7	11	3	8	7242.1196	-0.0066
10	5	7	12	4	8	7746.9645	-0.0022

D_{15}-D_{16}

J′	K_{-1}′	K_{+1}′	J′′	K_{-1}′′	K_{+1}′′	Obs.	Obs.-Cal.
3	1	3	2	0	2	6032.8116	-0.0159
3	2	2	3	1	3	5159.8023	0.0082
4	0	4	3	1	3	6694.6938	0.0088
4	1	4	3	0	3	7386.0638	0.0030
4	1	3	4	0	4	4221.9313	-0.0046
5	1	4	5	0	5	5873.0339	-0.0080
5	2	3	5	1	4	3764.1728	0.0067
5	2	4	5	1	5	7165.5617	0.0043
6	1	5	6	0	6	7662.0479	0.0130
6	2	4	6	1	5	4770.6545	0.0000
6	3	3	6	2	4	4825.3072	0.0053
7	2	5	7	1	6	6256.1982	-0.0038
7	3	4	7	2	5	4867.8606	-0.0004
8	3	5	8	2	6	5431.8520	0.0091
9	3	6	9	2	7	6573.0172	-0.0102
9	4	5	9	3	6	6364.2018	-0.0093
10	4	6	10	3	7	6404.5960	-0.0034
11	4	7	11	3	8	7054.7648	-0.0025

Table S20. Observed rotational transitions and residuals (all the values in MHz) for the conformer I-w-a of the salicylic acid – water complex in the ground vibrational state. The lines with frequencies above 8 GHz were measured in the MB-FTMW spectrometer as a thermal recombination product in the spectroscopic analysis of *o*-anisic acid [36].

J′	K_{-1}′	K_{+1}′	J′′	K_{-1}′′	K_{+1}′′	Obs.	Obs.-Cal.	J′	K_{-1}′	K_{+1}′	J′′	K_{-1}′′	K_{+1}′′	Obs.	Obs.-Cal.
1	1	1	0	0	0	2820.3825	-0.0004	5	1	4	5	1	5	6300.6709	0.0085
2	1	1	1	1	0	2440.2790	-0.0016	5	1	3	5	1	4	4493.7493	0.0048
2	0	2	1	0	1	2291.7953	-0.0019	6	2	6	5	1	5	6421.4530	0.0057
2	1	2	1	0	1	3830.4594	-0.0001	6	2	6	5	0	5	6634.0042	-0.0064
2	2	0	1	1	1	7599.5574	0.0041	6	1	5	5	1	4	7244.1329	-0.0002
2	2	0	2	1	1	5019.2293	-0.0009	6	0	4	5	2	3	7129.1423	0.0026
2	2	1	2	1	2	5430.9205	0.0052	6	1	5	5	2	4	6862.8118	0.0059
3	0	3	2	0	2	3416.7925	-0.0001	6	2	3	5	3	2	6956.2422	0.0045
3	1	3	2	1	2	3235.1704	0.0027	6	2	4	5	3	3	6938.6901	0.0088
3	1	2	2	1	1	3654.9774	0.0025	6	3	2	5	4	1	6929.6329	0.0065
3	2	1	2	2	0	3483.9167	-0.0023	6	3	6	5	1	5	5713.9422	-0.0053
3	2	2	2	2	1	3450.3533	-0.0010	6	4	6	5	0	5	7341.5124	0.0020
3	1	3	2	0	2	4773.8281	-0.0019	6	0	5	6	0	6	3621.9393	-0.0007
3	2	2	2	1	1	8461.1408	-0.0021	6	1	4	6	1	5	4378.7504	-0.0005
3	1	2	3	0	3	2196.9807	0.0094	6	1	5	6	1	6	6742.0017	-0.0193
3	2	1	3	1	2	4848.1764	0.0020	7	2	7	6	1	6	7468.3652	-0.0045
3	2	2	3	1	3	5646.0997	-0.0021	7	2	7	6	0	6	7653.9541	-0.0010
4	1	4	3	1	3	4304.4180	-0.0042	7	1	6	6	1	5	8409.7402	0.0002
4	0	4	3	0	3	4517.8419	-0.0029	7	0	5	6	2	4	8381.2270	-0.0015
4	1	3	3	1	2	4862.6841	0.0030	7	1	6	6	2	5	7985.5556	-0.0030
4	2	2	3	2	1	4676.5230	0.0048	7	2	7	6	0	6	8175.8719	0.0024
4	2	3	3	2	2	4593.8994	0.0007	7	2	7	6	1	6	6946.4605	0.0049
4	3	1	3	3	0	4618.3385	-0.0026	7	1	6	6	2	5	4582.1597	0.0003
4	3	2	3	3	1	4616.4323	-0.0009	7	0	6	7	0	7	4377.7273	0.0025
4	0	4	3	1	3	3160.8209	0.0134	7	1	5	7	1	6	4350.2385	-0.0009
4	1	4	3	0	3	5661.4550	-0.0046	7	1	6	7	1	7	7259.2296	0.0196
4	1	3	4	0	4	2541.8052	-0.0023	8	2	8	7	1	7	8507.8562	-0.0005
4	2	2	4	1	3	4662.0206	0.0090	8	2	8	7	0	7	8658.3758	-0.0004
4	2	3	4	1	4	5935.5789	0.0005	8	1	7	7	1	6	9552.1978	0.0006
5	1	5	4	1	4	5366.7482	0.0010	8	0	6	7	2	5	9638.2595	-0.0022
5	0	5	4	0	4	5590.2975	-0.0014	8	1	7	7	2	6	9098.9641	0.0027
5	1	4	4	1	3	6060.3106	0.0016	8	2	5	7	3	4	9342.0747	0.0011
5	2	3	4	2	2	5892.0385	-0.0034	8	2	8	7	0	7	9029.7704	-0.0006
5	2	4	4	2	3	5731.8269	-0.0042	8	3	7	7	2	6	6148.8009	0.0030
5	3	2	4	3	1	5783.0340	0.0001	8	1	7	8	0	8	5271.5353	-0.0103
5	3	3	4	3	2	5776.3927	0.0032	8	2	6	8	1	7	4436.3036	-0.0004
5	4	2	4	4	1	5769.3462	-0.0173	9	1	9	8	1	8	9540.6364	-0.0017
5	4	1	4	4	0	5769.3462	-0.0173	9	0	9	8	0	8	9655.5155	-0.0001
5	1	5	4	1	4	4446.6858	0.0017	9	1	8	9	0	9	6282.6835	0.0073
5	0	5	4	0	4	6510.3587	-0.0031	9	2	7	9	1	8	4659.5542	0.0030
5	0	4	5	0	5	3011.8175	-0.0001	10	2	8	10	1	9	5037.7868	-0.0122
								13	3	10	12	4	9	4662.8314	0.0009
								16	3	13	16	2	14	7409.6071	-0.0005

Table S21. Observed rotational transitions and residuals (all the values in MHz) for the D isotopomer of conformer I-w-a of salicylic acid – water complex in the ground vibrational state.

D_{15}

J'	K_{-1}'	K_{+1}'	J''	K_{-1}''	K_{+1}''	Obs.	Obs.-Cal.
5	1	5	4	0	4	6463.1357	0.0004
5	1	4	4	1	3	6004.3561	-0.0049
5	2	4	5	1	5	6265.9873	-0.0081
6	0	6	5	0	5	6576.5343	-0.0018
6	1	6	5	1	5	6365.2345	0.0013
6	1	5	5	1	4	7177.6381	-0.0125
6	2	4	5	2	3	7061.7961	-0.0002
6	2	5	5	2	4	6800.7922	-0.0073
6	3	3	5	3	2	6892.1394	0.0101
6	3	4	5	3	3	6875.1162	0.0142
6	2	5	6	1	6	6701.5700	0.0081
7	0	7	6	0	6	7588.2459	-0.0029
7	1	7	6	1	6	7403.2308	0.0054

D_{19}

J'	K_{-1}'	K_{+1}'	J''	K_{-1}''	K_{+1}''	Obs.	Obs.-Cal.
5	1	5	4	0	4	6413.6422	-0.0075
6	0	6	5	0	5	6470.7299	-0.0074
6	1	6	5	1	5	6259.9945	-0.0039
6	1	5	5	1	4	7039.3161	-0.0104
6	2	4	5	2	3	6915.0497	-0.0038
6	2	5	5	2	4	6676.2366	0.0077
6	3	3	5	3	2	6758.5973	0.0155
6	1	6	5	0	5	7225.0841	0.0090
6	2	5	6	1	6	6693.1619	0.0005
7	0	7	6	0	6	7469.9648	-0.0110
7	1	7	6	1	6	7282.2478	0.0107

D_{16}

J'	K_{-1}'	K_{+1}'	J''	K_{-1}''	K_{+1}''	Obs.	Obs.-Cal.
5	1	5	4	0	4	6442.8915	-0.0007
5	1	4	4	1	3	6051.2472	-0.0023
5	2	4	5	1	5	6186.9671	0.0023
6	0	6	5	0	5	6606.7178	0.0040
6	1	6	5	1	5	6397.7968	-0.0077
6	1	5	5	1	4	7231.4286	-0.0124
6	2	4	5	2	3	7125.6885	0.0014
6	2	5	5	2	4	6846.3920	0.0076
6	3	3	5	3	2	6945.5148	-0.0022
6	3	4	5	3	3	6926.2226	-0.0005
6	1	6	5	0	5	7270.7137	-0.0018
7	1	7	6	1	6	7439.7925	0.0046
7	0	7	6	1	6	6955.7608	0.0077

D_{18}

J'	K_{-1}'	K_{+1}'	J''	K_{-1}''	K_{+1}''	Obs.	Obs.-Cal.
6	0	6	5	0	5	6542.9574	-0.0015
6	1	6	5	1	5	6331.3803	-0.0036
6	1	5	5	1	4	7131.8099	0.0004
6	2	4	5	2	3	7012.0933	-0.0076
6	2	5	5	2	4	6759.8302	-0.0096
6	3	4	5	3	3	6831.5462	0.0121
7	0	7	6	0	6	7551.0337	0.0124
7	1	7	6	1	6	7364.4220	-0.0102
7	3	5	6	3	4	7977.2003	0.0038
7	0	7	6	1	6	6821.1920	0.0024

Table S22. Observed rotational transitions and residuals (all the values in MHz) for the I-w$_2$-a conformer of the salicylic acid –water$_2$ complex in the ground vibrational state.

J'	K_{-1}'	K_{+1}'	J''	K_{-1}''	K_{+1}''	Obs.	Obs.-Cal.	J'	K_{-1}'	K_{+1}'	J''	K_{-1}''	K_{+1}''	Obs.	Obs.-Cal.
2	1	2	1	0	1	2850.3823	0.0019	6	1	6	5	1	5	4562.0037	0.0006
2	2	0	1	1	1	5765.3289	-0.0012	6	0	6	5	0	5	4719.5048	-0.0084
2	2	0	2	1	1	3961.1660	0.0145	6	1	5	5	1	4	5099.4890	-0.0009
2	2	1	2	1	2	4229.2841	0.0000	6	2	4	5	2	3	4994.3497	0.0022
3	0	3	2	0	2	2415.5219	-0.0010	6	2	5	5	2	4	4846.8063	0.0048
3	1	3	2	1	2	2294.4946	0.0008	6	3	3	5	3	2	4896.1995	0.0114
3	1	2	2	1	1	2567.0230	0.0008	6	3	4	5	3	3	4888.3418	0.0051
3	1	3	2	0	2	3527.0104	-0.0021	6	4	2	5	4	1	4882.7053	0.0220
3	2	1	2	1	2	6685.4909	-0.0009	6	4	3	5	4	2	4882.5404	-0.0109
3	2	2	2	1	1	6390.2091	0.0002	6	5	1	5	5	0	4878.5206	0.0129
3	2	1	3	1	2	3845.7993	-0.0001	6	5	2	5	5	1	4878.5206	0.0129
3	2	2	3	1	3	4368.3789	-0.0064	6	1	6	5	0	5	5369.6333	0.0030
3	3	1	3	2	2	6824.3537	0.0075	6	1	5	6	0	6	2547.0057	0.0054
4	0	4	3	0	3	3200.0903	0.0024	6	2	4	6	1	5	3481.1254	0.0048
4	1	4	3	1	3	3054.3539	0.0028	6	2	5	6	1	6	5075.6521	0.0010
4	1	3	3	1	2	3417.0610	-0.0006	6	3	3	6	2	4	6611.7643	0.0066
4	2	2	3	2	1	3285.9668	0.0007	6	3	4	6	2	5	6902.2914	-0.0137
4	2	3	3	2	2	3241.2597	-0.0012	7	1	7	6	1	6	5309.0884	0.0037
4	1	4	3	0	3	4165.8406	-0.0001	7	0	7	6	0	6	5454.5611	-0.0019
4	2	2	3	1	3	7676.9481	-0.0158	7	1	6	6	1	5	5927.8315	0.0055
4	2	3	3	1	2	7064.4551	0.0075	7	2	5	6	2	4	5866.6237	-0.0006
4	2	2	4	1	3	3714.6987	-0.0049	7	2	6	6	2	5	5643.2330	-0.0060
4	2	3	4	1	4	4555.2847	-0.0103	7	3	4	6	3	3	5725.1791	0.0033
4	3	2	4	2	3	6836.5240	-0.0125	7	3	5	6	3	4	5707.6929	-0.0003
5	0	5	4	0	4	3968.4742	-0.0017	7	4	3	6	4	2	5701.2278	0.0125
5	1	5	4	1	4	3810.3516	0.0012	7	4	4	6	4	3	5700.7861	0.0092
5	1	4	4	1	3	4261.8374	0.0001	7	5	2	6	5	1	5694.4502	-0.0125
5	2	3	4	2	2	4133.3939	-0.0028	7	5	3	6	5	2	5694.4502	-0.0125
5	2	4	4	2	3	4045.9171	0.0093	7	0	7	6	1	6	4804.4569	0.0109
5	3	2	4	3	1	4073.1045	0.0005	7	1	7	6	0	6	5959.2098	0.0081
5	3	3	4	3	2	4070.1408	-0.0005	7	1	6	6	2	5	2749.0564	-0.0016
5	0	5	4	1	4	3002.7235	0.0003	7	1	6	7	0	7	3020.2674	0.0042
5	1	5	4	0	4	4776.1030	-0.0001	7	2	5	7	1	6	3419.9169	-0.0020
5	2	4	4	1	3	7693.2798	-0.0137	7	2	6	7	1	7	5409.8099	0.0044
5	2	3	5	1	4	3586.2642	0.0010	7	3	4	7	2	5	6470.3153	0.0061
5	2	4	5	1	5	4790.8594	0.0068	8	1	8	7	1	7	6051.6304	0.0015
5	3	2	5	2	3	6709.9106	-0.0066	8	0	8	7	0	7	6177.2483	-0.0108
5	3	3	5	2	4	6860.7894	0.0192	8	1	7	7	1	6	6744.3329	0.0026

Table S22 (Continued).

J′	K-1′	K+1′	J′′	K-1′′	K+1′′	Obs.	Obs.-Cal.	J′	K-1′	K+1′	J′′	K-1′′	K+1′′	Obs.	Obs.-Cal.
8	2	6	7	2	5	6745.6801	-0.0099	10	1	9	9	2	8	6072.9160	0.0175
8	2	7	7	2	6	6434.5679	-0.0002	10	1	9	10	0	10	4968.8030	0.0078
8	3	5	7	3	4	6561.9892	-0.0171	10	2	8	10	1	9	3671.3362	-0.0116
8	3	6	7	3	5	6527.6079	-0.0011	10	3	7	10	2	8	5835.2668	-0.0088
8	4	4	7	4	3	6522.1184	-0.0083	11	1	11	10	1	10	8255.4204	0.0027
8	4	5	7	4	4	6520.9338	0.0067	11	1	10	10	2	9	7170.4743	-0.0042
8	5	3	7	5	2	6511.7363	-0.0108	11	1	10	11	0	11	5751.2823	-0.0073
8	5	4	7	5	3	6511.7363	-0.0108	11	2	10	11	1	11	7215.8052	0.0054
8	0	8	7	1	7	5672.6052	-0.0153	12	3	9	12	2	10	5404.6643	0.0173
8	1	8	7	0	7	6556.2806	0.0130	13	1	12	13	0	13	7401.9730	0.0061
8	1	7	8	0	8	3587.3315	-0.0026	13	3	10	13	2	11	5257.4606	-0.0043
8	3	5	8	2	6	6286.6271	0.0016	15	3	12	15	2	13	5209.8231	0.0012
8	3	6	8	2	7	7059.8040	0.0036								
9	1	9	8	1	8	6789.8926	-0.0027								
9	0	9	8	0	8	6892.2673	0.0015								
9	1	8	8	1	7	7546.3109	0.0032								
9	2	7	8	2	6	7625.8658	0.0024								
9	2	8	8	2	7	7220.2226	0.0073								
9	3	6	8	3	5	7408.8017	0.0110								
9	3	7	8	3	6	7347.2530	-0.0049								
9	4	5	8	4	4	7345.9653	-0.0128								
9	4	6	8	4	5	7343.1224	0.0033								
9	5	5	8	5	4	7330.5143	-0.0117								
9	6	3	8	6	2	7323.2790	0.0030								
9	6	4	8	6	3	7323.2790	0.0030								
9	0	9	8	1	8	6513.2612	0.0037								
9	1	9	8	0	8	7168.8924	-0.0111								
9	1	8	8	2	7	4961.8785	-0.0103								
9	1	8	9	0	9	4241.3820	0.0058								
9	2	7	9	1	8	3500.8181	-0.0162								
9	2	8	9	1	9	6223.0563	-0.0084								
10	0	10	9	0	9	7603.8071	0.0011								
10	1	10	9	1	9	7524.3052	-0.0086								
10	1	9	9	1	8	8331.2251	0.0001								
10	2	8	9	2	7	8501.7360	-0.0024								
10	2	9	9	2	8	7999.7196	0.0031								
10	0	10	9	1	9	7327.1710	0.0029								
10	1	10	9	0	9	7800.9495	-0.0022								

Table S23. Observed rotational transitions and residuals (all the values in MHz) for the conformer I-w3-a of the salicylic acid –water2 complex in the ground (v=0) and first (v=1) vibrational states.

J′	K_{-1}′	K_{+1}′	J′′	K_{-1}′′	K_{+1}′′	v	Obs.	Obs.-Cal.	J′	K_{-1}′	K_{+1}′	J′′	K_{-1}′′	K_{+1}′′	v	Obs.	Obs.-Cal.
2	2	1	1	1	0	0	4498.0969	-0.0030	5	3	2	5	2	3	0	5569.8954	0.0108
2	2	1	1	1	0	1	4498.0969	-0.0030	6	1	6	5	1	5	0	3279.5986	-0.0027
3	2	1	2	1	2	0	5194.9698	-0.0185	6	1	6	5	1	5	1	3279.7263	0.0020
3	2	1	2	1	2	1	5194.9004	-0.0005	6	1	5	5	1	4	0	3607.3740	-0.0128
3	2	2	2	1	1	1	5019.6712	0.0028	6	1	5	5	1	4	1	3607.4512	-0.0131
3	2	2	2	1	1	0	5019.6712	0.0028	6	2	4	5	2	3	0	3518.5406	-0.0033
3	3	0	2	2	1	0	7351.8188	-0.0010	6	2	5	5	2	4	0	3450.6241	-0.0070
3	3	0	2	2	1	1	7351.6907	0.0180	6	2	5	5	2	4	1	3450.6930	-0.0171
3	3	1	2	2	0	0	7349.7515	0.0002	6	3	3	5	3	2	0	3472.2202	0.0047
3	3	1	2	2	0	1	7349.6119	0.0073	6	3	3	5	3	2	1	3472.2202	0.0047
3	2	1	3	1	2	0	3218.6146	-0.0045	6	1	6	5	0	5	1	4047.1374	0.0061
3	2	1	3	1	2	1	3218.5337	0.0074	6	2	4	6	1	5	0	2949.1094	-0.0035
3	2	2	3	1	3	0	3538.8605	-0.0112	6	2	5	6	1	6	0	3963.9175	-0.0018
3	2	2	3	1	3	1	3538.7372	0.0022	6	2	5	6	1	6	1	3963.7312	-0.0066
3	3	0	3	2	1	0	5611.8717	0.0113	6	3	3	6	2	4	0	5523.5388	0.0192
3	3	0	3	2	1	1	5611.6807	0.0033	6	3	3	6	2	4	1	5523.3437	-0.0125
4	0	4	3	0	3	0	2286.4552	0.0042	7	0	7	6	0	6	0	3931.0615	-0.0017
4	0	4	3	0	3	1	2286.4552	0.0042	7	0	7	6	0	6	1	3931.1871	0.0110
4	1	4	3	0	3	0	3160.2814	0.0026	7	1	7	6	1	6	0	3819.8589	-0.0009
4	1	4	3	0	3	1	3160.2814	0.0026	7	1	7	6	1	6	1	3819.9899	0.0057
4	2	3	4	1	4	0	3651.3358	-0.0063	7	1	6	6	1	5	0	4199.5088	-0.0162
4	2	3	4	1	4	1	3651.1854	0.0055	7	1	6	6	1	5	1	4199.5876	-0.0097
4	3	2	4	2	3	0	5627.3813	-0.0137	7	2	5	6	2	4	0	4125.8391	0.0015
4	3	2	4	2	3	1	5627.2080	-0.0003	7	2	5	6	2	4	1	4125.8391	0.0015
5	0	5	4	0	4	0	2843.4585	0.0010	7	3	4	6	3	3	0	4056.1818	0.0019
5	0	5	4	0	4	1	2843.5408	0.0103	7	3	4	6	3	3	1	4056.1818	0.0019
5	1	5	4	1	4	0	2737.0847	-0.0007	7	3	5	6	3	4	0	4050.3505	-0.0022
5	1	5	4	1	4	1	2737.0847	-0.0007	7	3	5	6	3	4	1	4050.4368	-0.0016
5	1	4	4	1	3	0	3011.4643	-0.0009	7	5	2	6	5	1	1	4043.6532	-0.0014
5	1	4	4	1	3	1	3011.4643	-0.0009	7	5	3	6	5	2	1	4043.6532	-0.0014
5	2	3	4	2	2	0	2918.2721	-0.0029	7	0	7	6	1	6	0	3275.8068	0.0079
5	2	3	4	2	2	1	2918.2721	-0.0029	7	0	7	6	1	6	1	3275.9063	-0.0003
5	2	4	4	2	3	0	2878.6130	0.0002	7	1	7	6	0	6	1	4475.2660	0.0124
5	2	4	4	2	3	1	2878.6730	-0.0044	7	2	5	7	1	6	0	2875.3619	-0.0008
5	1	5	4	0	4	0	3610.9246	0.0109	7	2	5	7	1	6	1	2875.3619	-0.0008
5	1	5	4	0	4	1	3610.9246	0.0109	7	2	6	7	1	7	0	4164.7058	0.0016
5	2	4	5	1	5	0	3792.8982	0.0085	7	2	6	7	1	7	1	4164.4981	0.0048
5	2	4	5	1	5	1	3792.7505	-0.0014	7	3	4	7	2	5	0	5453.8508	-0.0047

Table S23 (Continued).

J'	K_{-1}'	K_{+1}'	J''	K_{-1}''	K_{+1}''	v	Obs.	Obs.-Cal.
7	3	4	7	2	5	1	5453.6964	-0.0083
7	3	5	7	2	6	0	5687.0288	-0.0132
7	3	5	7	2	6	1	5686.8413	0.0028
8	0	8	7	0	7	0	4462.0436	0.0035
8	0	8	7	0	7	1	4462.1763	0.0013
8	1	8	7	1	7	0	4357.7494	-0.0003
8	1	8	7	1	7	1	4357.8974	0.0061
8	2	6	7	2	5	0	4738.9407	-0.0021
8	2	6	7	2	5	1	4739.0235	-0.0022
8	2	7	7	2	6	0	4588.3474	0.0062
8	2	7	7	2	6	1	4588.4661	0.0091
8	3	5	7	3	4	0	4643.1704	-0.0048
8	3	5	7	3	4	1	4643.2797	0.0108
8	3	6	7	3	5	0	4631.7135	0.0092
8	3	6	7	3	5	1	4631.8139	0.0121
8	0	8	7	1	7	0	3917.9794	0.0002
8	0	8	7	1	7	1	3918.0947	-0.0027
8	1	8	7	0	7	0	4901.8099	-0.0007
8	1	8	7	0	7	1	4901.9630	-0.0057
8	2	6	8	1	7	0	2827.5036	-0.0112
8	2	6	8	1	7	1	2827.5036	-0.0112
8	2	7	8	1	8	0	4395.3123	0.0168
8	2	7	8	1	8	1	4395.0584	-0.0005
8	3	5	8	2	6	0	5358.0903	0.0023
8	3	5	8	2	6	1	5357.9308	-0.0171
9	0	9	8	0	8	0	4986.2897	0.0023
9	0	9	8	0	8	1	4986.4408	-0.0039
9	1	9	8	1	8	0	4893.2757	-0.0034
9	1	9	8	1	8	1	4893.4389	-0.0008
9	1	8	8	1	7	0	5368.0884	0.0068
9	1	8	8	1	7	1	5368.1716	-0.0078
9	2	7	8	2	6	0	5355.9481	0.0024
9	2	7	8	2	6	1	5356.0761	-0.0013
9	2	8	8	2	7	0	5153.4320	0.0033
9	2	8	8	2	7	1	5153.6041	0.0011
9	3	6	8	3	5	1	5234.2430	-0.0083
9	3	7	8	3	6	0	5213.3825	-0.0039
9	3	7	8	3	6	1	5213.4955	-0.0002
9	4	6	8	4	5	0	5208.7158	-0.0001
9	4	6	8	4	5	1	5208.8316	0.0059
9	0	9	8	1	8	0	4546.5049	-0.0118
9	0	9	8	1	8	1	4546.6503	-0.0005
9	1	9	8	0	8	0	5333.0471	-0.0027
9	1	9	8	0	8	1	5333.2402	0.0066
10	0	10	9	0	9	0	5505.8622	-0.0075
10	0	10	9	0	9	1	5506.0474	-0.0020
10	1	10	9	1	9	0	5426.6261	-0.0086
10	1	10	9	1	9	1	5426.6261	-0.0086
10	1	9	9	1	8	0	5942.2026	0.0006
10	1	9	9	1	8	1	5942.3107	-0.0056
10	3	7	9	3	6	0	5829.9987	0.0025
10	3	7	9	3	6	1	5830.1140	0.0077
10	0	10	9	1	9	0	5159.1060	-0.0012
10	0	10	9	1	9	1	5159.2556	-0.0050
10	1	10	9	0	9	0	5773.3106	0.0034
10	1	9	9	1	8	0	5942.2023	0.0004
10	1	9	9	1	8	1	5942.3102	-0.0061
10	3	7	9	3	6	0	5829.9982	0.0020
10	3	7	9	3	6	1	5830.1138	0.0075
11	0	11	10	0	10	0	6022.8168	0.0003
11	0	11	10	0	10	1	6023.0202	0.0020
11	1	11	10	1	10	0	5957.7224	0.0083
11	1	11	10	1	10	1	5957.9005	-0.0135
11	1	10	10	1	9	0	6507.9718	0.0042
11	1	10	10	1	9	1	6508.0954	-0.0052
11	2	9	10	2	8	0	6591.6859	0.0041
11	3	8	10	3	7	1	6431.7558	0.0035
11	3	9	10	3	8	0	6376.2692	-0.0048
11	3	9	10	3	8	1	6376.4084	-0.0003
11	0	11	10	1	10	1	5755.5525	-0.0015
11	1	11	10	0	10	0	6225.1607	0.0092
11	1	11	10	0	10	1	6225.3710	-0.0072
12	0	12	11	0	11	1	6538.9550	-0.0096
12	0	12	11	0	11	0	6538.7405	-0.0009
12	1	12	11	1	11	0	6487.0078	0.0020
12	1	12	11	1	11	1	6487.2333	-0.0006
12	2	10	11	2	9	1	7205.7509	-0.0006
12	2	11	11	2	10	0	6830.6052	-0.0017
12	2	11	11	2	10	1	6830.7892	-0.0004
12	1	12	11	0	11	1	6689.5912	0.0051
13	0	13	12	0	12	0	7054.7088	0.0024
13	0	13	12	0	12	1	7054.9563	0.0058
13	1	12	12	1	11	1	7610.9945	-0.0049

Table S24. Observed rotational transitions and residuals (all the values in MHz) for the conformer I-w4-a of the salicylic acid –water2 complex in the ground vibrational state.

J´	K_{-1}´	K_{+1}´	J´´	K_{-1}´´	K_{+1}´´	Obs.	Obs.-Cal.	J´	K_{-1}´	K_{+1}´	J´´	K_{-1}´´	K_{+1}´´	Obs.	Obs.-Cal.
3	1	3	2	0	2	2146.8089	-0.0062	7	1	7	6	0	6	3751.3734	0.0006
3	3	0	2	2	1	5513.9673	0.0132	7	2	6	6	1	5	5460.7957	-0.0084
3	3	1	2	2	0	5513.2546	-0.0077	7	3	4	6	2	5	7409.6071	-0.0009
4	1	4	3	0	3	2562.6496	-0.0015	7	3	5	6	2	4	7360.3244	0.0022
4	3	1	3	2	2	5984.4313	0.0017	7	3	4	7	2	5	4047.5752	0.0048
4	3	2	3	2	1	5980.9664	0.0137	7	3	5	7	2	6	4129.1939	0.0114
4	3	1	4	2	2	4098.4762	-0.0011	8	0	8	7	0	7	3698.5373	0.0051
4	3	2	4	2	3	4108.5900	-0.0009	8	1	8	7	1	7	3630.9163	0.0220
5	0	5	4	0	4	2331.8498	-0.0033	8	1	7	7	1	6	3846.7699	-0.0109
5	1	4	4	1	3	2410.5358	-0.0024	8	2	6	7	2	5	3798.1278	0.0129
5	2	3	4	2	2	2357.3344	-0.0054	8	2	7	7	2	6	3744.2092	0.0129
5	2	4	4	2	3	2343.8449	-0.0008	8	3	5	7	3	4	3762.0754	0.0030
5	1	5	4	0	4	2967.3543	0.0025	8	3	6	7	3	5	3759.4316	0.0054
5	3	2	4	2	3	6456.4877	-0.0052	8	5	3	7	5	2	3755.9425	0.0005
5	3	3	4	2	2	6446.0107	0.0027	8	5	4	7	5	3	3755.9425	0.0005
5	3	2	5	2	3	4088.9710	0.0058	8	0	8	7	1	7	3193.7919	0.0066
5	3	3	5	2	4	4112.3457	-0.0043	8	1	8	7	0	7	4135.6564	-0.0137
6	0	6	5	0	5	2791.0225	-0.0003	8	2	7	7	1	6	5835.5772	0.0037
6	1	6	5	1	5	2727.3138	-0.0008	8	3	5	7	2	6	7893.4910	-0.0019
6	1	5	5	1	4	2890.5948	0.0004	8	4	5	7	4	4	3757.3312	0.0055
6	2	4	5	2	3	2834.7346	0.0015	8	3	5	8	2	6	4011.4997	-0.0006
6	2	5	5	2	4	2811.3499	-0.0051	8	3	6	8	2	7	4144.4075	-0.0060
6	3	3	5	3	2	2818.4821	-0.0011	9	0	9	8	0	8	4146.8516	0.0033
6	3	4	5	3	3	2817.8846	-0.0056	9	1	9	8	1	8	4081.2990	-0.0003
6	1	6	5	0	5	3362.8182	0.0049	9	1	8	8	1	7	4322.2770	0.0125
6	3	3	5	2	4	6931.1303	-0.0001	9	2	7	8	2	6	4283.9072	0.0092
6	3	4	5	2	3	6906.5580	-0.0004	9	2	8	8	2	7	4209.3349	-0.0125
6	3	3	6	2	4	4072.7183	0.0028	9	3	6	8	3	5	4235.4439	-0.0125
6	3	4	6	2	5	4118.8850	-0.0002	9	3	7	8	3	6	4230.6017	0.0109
7	0	7	6	0	6	3246.6293	-0.0033	9	4	5	8	4	4	4228.2264	0.0081
7	1	7	6	1	6	3179.5783	0.0001	9	4	6	8	4	5	4228.1088	-0.0016
7	1	6	6	1	5	3369.4318	-0.0004	9	5	4	8	5	3	4226.1287	-0.0010
7	2	5	6	2	4	3314.9854	-0.0013	9	5	5	8	5	4	4226.1287	-0.0010
7	2	6	6	2	5	3278.1774	-0.0021	9	6	3	8	6	2	4225.0956	-0.0054
7	3	4	6	3	3	3289.8309	-0.0014	9	6	4	8	6	3	4225.0956	-0.0054
7	3	5	6	3	4	3288.5017	0.0002	9	0	9	8	1	8	3709.7226	0.0015
7	4	3	6	4	2	3286.9172	0.0061	9	1	9	8	0	8	4518.4211	-0.0108
7	4	4	6	4	3	3286.9172	0.0061	9	2	8	8	1	7	6198.1354	0.0026

Table S24 (Continued).

J′	K-1′	K+1′	J′′	K-1′′	K+1′′	Obs.	Obs.-Cal.
9	3	6	9	2	7	3963.0512	0.0112
10	0	10	9	0	9	4591.9952	-0.0021
10	1	10	9	1	9	4530.6937	-0.0062
10	1	9	9	1	8	4795.6070	0.0067
10	2	8	9	2	7	4771.7779	0.0020
10	2	9	9	2	8	4673.4526	-0.0004
10	3	7	9	3	6	4710.2044	0.0091
10	3	8	9	3	7	4701.9874	0.0108
10	4	6	9	4	5	4699.4698	-0.0114
10	4	7	9	4	6	4699.2522	0.0050
10	5	5	9	5	4	4696.5744	0.0115
10	5	6	9	5	5	4696.5744	0.0115
10	6	4	9	6	3	4695.1329	-0.0033
10	6	5	9	6	4	4695.1329	-0.0033
10	0	10	9	1	9	4220.4285	-0.0014
10	1	10	9	0	9	4902.2522	-0.0152
10	3	7	10	2	8	3901.4668	0.0073
10	3	8	10	2	9	4194.2036	-0.0096
11	0	11	10	0	10	5034.5708	-0.0035
11	1	11	10	1	10	4979.1321	-0.0017
11	1	10	10	1	9	5266.3543	0.0010
11	2	9	10	2	8	5260.9964	0.0038
11	2	10	10	2	9	5136.4677	-0.0014
11	3	8	10	3	7	5186.6494	0.0026
11	3	9	10	3	8	5173.4379	-0.0015
11	4	7	10	4	6	5171.2630	0.0105
11	4	8	10	4	7	5170.7828	-0.0029
11	5	6	10	5	5	5167.2611	-0.0080
11	5	7	10	5	6	5167.2611	-0.0080
11	7	4	10	7	3	5164.2731	0.0083
11	7	5	10	7	4	5164.2731	0.0083
11	0	11	10	1	10	4724.2990	-0.0053
11	1	11	10	0	10	5289.4113	0.0073
11	2	10	10	1	9	6890.1814	-0.0052
11	3	9	11	2	10	4231.1812	-0.0023
12	0	12	11	0	11	5475.2903	-0.0060
12	1	12	11	1	11	5426.6261	-0.0103
12	1	11	11	1	10	5734.1331	0.0074
12	2	10	11	2	9	5750.6893	0.0019
12	2	11	11	2	10	5598.2924	-0.0016
12	3	9	11	3	8	5665.1083	-0.0006
12	3	10	11	3	9	5644.8739	0.0048
12	4	8	11	4	7	5643.5959	-0.0132
12	4	9	11	4	8	5642.7359	-0.0019
12	5	7	11	5	6	5638.2794	0.0031
12	5	8	11	5	7	5638.2794	0.0031

J′	K-1′	K+1′	J′′	K-1′′	K+1′′	Obs.	Obs.-Cal.
12	0	12	11	1	11	5220.4656	-0.0011
12	1	12	11	0	11	5681.4625	-0.0034
12	2	11	11	1	10	7222.1241	-0.0034
13	0	13	12	0	12	5914.8523	0.0005
13	1	13	12	1	12	5873.2651	0.0029
13	1	12	12	1	11	6198.5138	-0.0009
13	2	11	12	2	10	6240.0009	0.0007
13	2	12	12	2	11	6058.8490	-0.0014
13	3	10	12	3	9	6145.8999	0.0077
13	3	11	12	3	10	6116.1345	0.0111
13	4	9	12	4	8	6116.6417	-0.0073
13	4	10	12	4	9	6115.0977	-0.0110
13	5	8	12	5	7	6109.6050	-0.0078
13	5	9	12	5	8	6109.6050	-0.0078
13	6	7	12	6	6	6106.4092	-0.0019
13	6	8	12	6	7	6106.4092	-0.0019
13	0	13	12	1	12	5708.6726	-0.0095
13	1	13	12	0	12	6079.4403	0.0085
13	1	12	12	2	11	4710.5208	0.0080
13	2	11	13	1	12	2117.0818	-0.0007
13	3	10	13	2	11	3647.4340	0.0065
14	0	14	13	0	13	6353.8201	0.0021
14	1	14	13	1	13	6319.0742	-0.0060
14	1	13	13	1	12	6659.1514	-0.0095
14	2	12	13	2	11	6728.1741	0.0125
14	2	13	13	2	12	6518.0813	0.0069
14	3	11	13	3	10	6629.2689	0.0079
14	3	12	13	3	11	6587.0546	0.0085
14	4	10	13	4	9	6590.5007	-0.0121
14	5	9	13	5	8	6581.2954	-0.0121
14	6	8	13	6	7	6577.2972	0.0104
14	6	9	13	6	8	6577.2972	0.0104
14	1	14	13	0	13	6483.6656	0.0054
15	0	15	14	0	14	6792.6297	0.0103
15	1	15	14	1	14	6764.1666	-0.0023
15	2	13	14	2	12	7214.5289	-0.0011
15	3	12	14	3	11	7115.3710	-0.0115
15	3	13	14	3	12	7057.4591	-0.0135
16	0	16	15	0	15	7231.5346	0.0059
16	1	16	15	1	15	7208.6075	-0.0039
16	1	15	15	1	14	7568.3080	-0.0055
16	5	11	15	5	10	7526.0149	-0.0085
16	5	12	15	5	11	7525.7635	0.0047
16	1	16	15	0	15	7310.0003	-0.0028
17	0	17	16	0	16	7670.6928	-0.0017
